# The Prediction of Survival in Hepatocellular Carcinoma Based on A Four Long Non-coding RNAs Expression Signature

**DOI:** 10.7150/jca.40621

**Published:** 2020-04-12

**Authors:** Zongxing Yang, Yuhan Yang, Gang Zhou, Yan Luo, Wenjun Yang, Youliang Zhou, Jin Yang

**Affiliations:** 1 The Second Department of Infectious Disease, Xixi Hospital of Hangzhou, the Affiliated Hospital of Zhejiang Chinese Medical University, Zhejiang Chinese Medical University, Hangzhou, Zhejiang 310023, P.R. China.; 2 Center for Translational Medicine, the affiliated hospital of Hangzhou Normal University, Hangzhou Normal University, Hangzhou, Zhejiang 310015, P.R. China.

**Keywords:** Hepatocellular carcinoma, Prognosis, lncRNA, Gene expression signature.

## Abstract

Prognostic stratification in hepatocellular carcinoma (HCC) patients is still challenging. Long non-coding RNAs (lncRNAs) have been proven to play a crucial role in tumorigenesis and progression of cancers. The aim of this study is to develop a useful prognostic index based on lncRNA signature to identify patients at high risk of disease progression. We obtained lncRNA expression profiles from three publicly available datasets from Gene Expression Omnibus (GEO) and The Cancer Genome Atlas (TCGA). By the risk scoring method, we built an individualized four-lncRNA signature (HCCLnc-4) to predict survival of HCC patients in the discovery set (ROC curve, AUC: 0.83, 95% CI: 0.65-1.00, *P* < 0.05, Kaplan-Meier analysis and log-rank test, *P* < 0.01). Similar prognostic value of HCCLnc-4 has been further verified in two other independent sets. Stratified analysis and multivariate Cox regression analysis suggested the independence of HCCLnc-4 for prediction of HCC patient survival from traditional clinicopathological factors. Area under curve (AUC) analysis suggested that HCCLnc-4 could compete sufficiently with, or might be even better than classical pathological staging systems to predict HCC patient prognosis in the same data sets. Functional analysis and network analysis suggested the potential implication of lncRNA biomarkers. Our study developed and validated the lncRNA prognostic index of HCC patients, warranting further clinical evaluation and preventive interventions.

## Introduction

Hepatocellular carcinoma (HCC), as the most common type of liver cancers, ranks as the fifth most common cancer globally and is the second most common cause of death by cancer worldwide 1. Accurately estimating HCC patients' prognosis, choosing effective treatment protocol for high-risk patients, and prolonging the survival time are greatly important in clinical practice. Previous studies suggested numerous factors related to the prognosis of HCC, including gender 2, age 3, 4, infection of HBV 5, 6, cirrhosis 7, alpha-fetoprotein (AFP) levels 8, 9, and various pathophysiological characteristics of tumor 8, 10. However, because of highly clinically and molecularly heterogeneity of human HCC, and hence our limited understanding of the mechanisms underlying tumorigenesis and development of HCC, evaluation based on these traditional prognosis factors is not comprehensive. Moreover, given the systematic measurement biases due to the experimental batch effects, a more personalized prognostic evaluation is needed for individual patients to guide clinical treatments 11-13. With the advance of high-resolution microarrays and sequencing technology, numerous tissue- and serum-associated HCC biomarkers as potential diagnostic, prognostic, and therapeutic targets were presented 14. Among them, one class of newly discovered non-coding RNAs (ncRNAs), long non-coding RNAs (lncRNAs), have obtained increasing attention 15.

lncRNAs are typically defined as non-coding transcripts longer than 200 nucleotides 16. Growing evidence indicates that lncRNAs are involved in all essential biological processes in living cells 17. In the context of HCC, recent studies suggested that by varying mechanisms, including splicing, differential expression, epigenetic silencing, lncRNA-protein interaction, and lncRNA-miRNA interaction, HCC-related lncRNAs participate in many processes involved in HCC pathogenesis, such as cell proliferation, apoptosis, angiogenesis, and metastasis 15. Recently, lncRNAs as molecular biomarkers for early detection, monitoring recurrence, prediction of survival, and prediction of treatment response of HCC, were reviewed by Doctor Mitsuro and colleagues 18. Some lncRNAs, such as lncRNA *MALAT1* 19, lncRNA *HOTAIR* 20, lncRNA *CASC15*, lncRNA *CCAT1*, lncRNA *CCAT2*, lncRNA *LASP1-AS*, lncRNA *LINC00673*, lncRNA *LOC90784*, lncRNA *NEAT1*, and lncRNA *SBF2-AS1* might act as prognostic biomarkers to predict HCC progression or recurrence 18.

In this study, from the perspective of lncRNA expression pattern, applying three datasets of gene expression profiles from Gene Expression Omnibus (GEO) and The Cancer Genome Atlas (TCGA) for 447 HCC patients, we investigated associations between expression levels of lncRNAs and survival outcomes of HCC patients. By the risk scoring method, we established and validated an individualized four-lncRNA signature in different datasets. Functional analysis suggested the four lncRNAs and related genes might be involved in known HCC-related pathophysiologic processes.

## Materials and Methods

### HCC patient cohorts

**Figure 1:**
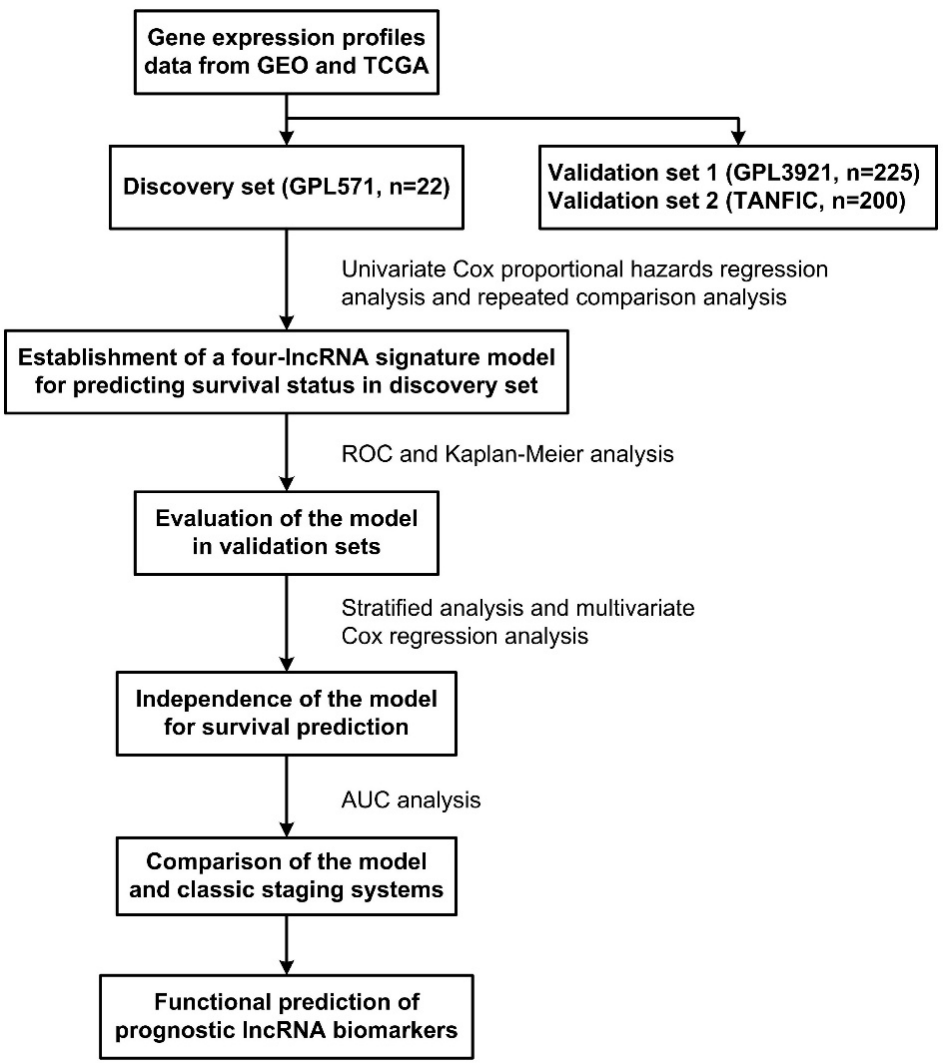
Diagram showing the process to build and validate the lncRNA signature risk score model to predict prognostic outcomes.

The GSE14520 (https://www.ncbi.nlm.nih.gov/geo/query/acc.cgi?acc=GSE14520) dataset contained two batches of samples. The smaller batch included 22 HCC samples, generated by Affymetrix Human Genome U133A 2.0 Array platform, were used to train a prognostic signature. The larger batch included 225 HCC samples, generated by Affymetrix HT Human Genome U133A Array, were used as the first validation dataset (test cohort-1). The second validation dataset was composed of 200 TCGA HCC samples from TANRIC database (TANRIC: An interactive open platform to explore the function of lncRNAs in cancer.), denoted as test cohort-2. All non-coding transcripts included in the three datasets were for patients treated with surgery only. In addition, the RNA and miRNA expression data, as well as the clinical data of HCC patients were obtained from TCGA (https://tcga-data.nci.nih.gov/tcga/). The flow chart of the study is described in Figure 1.

### Probe re-annotation and identification of lncRNA

The probe sets ID represented on the Affymetrix microarrays were checked by NetAffx Annotation Files (HG-U133A_2 Annotations, CSV format, Release 36, 19 MB, 4/13/16; HT_HG-U133A Annotations, CSV format, Release 36, 19 MB, 4/13/16), obtained from the Affymetrix official website. BioMart in Ensembl database was applied to convert Affymetrix microarray IDs to Ensembl IDs together with the corresponding gene type. We only retained genes annotated as 'lincRNA', 'sense_intronic', 'processed_transcript', 'anti-sense', 'sense_overlapping', '3prime_overlapping_ncrna', or 'misc_RNA'. Next, for the probe sets from the Refseq database, those IDs beginning with 'NR' were retained, and transcript IDs labeled with 'NP' were deleted. We removed probe set IDs annotated as 'microRNA', 'snoRNAs', ' pseudo-genes' and other small RNAs. Finally, 842 lncRNA-specific probes corresponding to 351 lncRNAs were obtained for further analysis. When multiple probes were mapped to the same lncRNA, expression values of these probes were integrated by using the median value to represent the expression value of the single lncRNAs. All of the raw data were processed using affy and related R packages with Robust Multi-array Average approach for background normalization as per the package instruction.

### Statistical analysis

The association between expression levels of lncRNAs and HCC patients' overall survival (OS) was evaluated by univariate Cox proportional hazards regression analysis (*P* < 0.001 as selection criteria). Then the combinations of lncRNAs related to HCC patients' OS were analyzed by repeated comparison analysis to identify the best prognostic model for predicting the OS of patients. Next, the lncRNA expression signature, termed HCCLnc-4, was established by the risk scoring method, as described by Lossos et al 21. Then the risk score value that produced the shortest distance to the point of perfect prediction of the ROC curve, was selected as the cutoff point. By the cutoff value that was determined by the ROC curve, patients were divided into low-risk and high-risk groups. Kaplan-Meier survival analysis and log-rank test were used to compare the difference in OS time between the high-risk group and low-risk group. Given other clinicopathological factors associated with HCC patient OS time as confounding variables, stratified analysis and multivariate Cox regression analysis were performed to evaluate the independence of the lncRNA expression signature to predict the OS outcomes of HCC patients. Area under curve (AUC) analysis was used to determine the superiority of HCCLnc-4 for prediction of HCC patient OS comparing with traditional clinicopathological staging systems. SPSS version 19.0 software (SPSS Inc., Chicago, IL) and GraphPad Prism 5.0 (GraphPad Software, San Diego, CA) was used for statistical analysis and graphics, respectively. The statistical significance level was 0.05.

### Functional enrichment analysis

Pearson correlation coefficients were calculated between lncRNAs and protein-coding genes (PCGs) or miRNAs. Functional enrichment analysis for the correlated PCGs was performed using DAVID Bioinformatics Tool (https://david.ncifcrf.gov/, version 6.7) 22. The network was generated by highly correlated lncRNAs-miRNAs-mRNAs and visualized by Cytoscape 3.2 and ggalluvial package of R software 23.

## Results

### Patient characteristics

Three HCC patient cohorts with definitive OS information were included in this study. The patient cohort from GSE14520 (GPL571 platform) was selected as a discovery cohort. The patient cohorts from GSE14520 (GPL3921 platform) and TCGA (TANRIC) were used as independent validation cohorts to verify the robustness of the lncRNA biomarkers (hereafter referred as test cohort-1, and test cohort-2, respectively). All 447 patients used in this study were clinically and pathologically diagnosed with HCC. The mean OS time was 31.8 months (range, 1.8-63.8 months, discovery cohort), 40.4 months (range, 2-67.4 months, test cohort-1), and 24.6 months (range, 0-115.9 months, test cohort-2), respectively. All the statistical information was summarized in Table 1 and Table S1.

### Identification of lncRNAs associated with OS of HCC patients from the discovery set

As shown in Figure 1, the discovery set was firstly analyzed to identify the potential prognostic lncRNA biomarkers, and then the validation data sets were conducted for validation. In the discovery set, univariate Cox proportional hazards regression analysis was performed for lncRNA expression data, and lncRNAs related to patient OS (*P* < 0.001 as selection criteria) were identified. Then the combinations of these lncRNAs related to patient OS were analyzed, and a model consisting of four lncRNAs was identified as the best prognostic model for predicting the OS of patients. All these four lncRNAs were verified in the NCBI database and classified as ncRNAs in this website. The detailed information of these four lncRNAs is shown in Table 2. Subsequently, these four lncRNAs were integrated into a predictive signature (hereafter inferred as HCCLnc-4) by risk scoring method that was described in “Materials and methods”, to predict the prognostic outcomes, as follows: HCCLnc-4 risk score = (3.6392 × expression value of ENSG00000234608) + (-2.9565 × expression value of ENSG00000242086) + (-6.9077 × expression value of ENSG00000273032) + (1.5738 × expression value of ENSG00000228463).

### Association of HCCLnc-4 and patient OS in the discovery set

**Figure 2:**
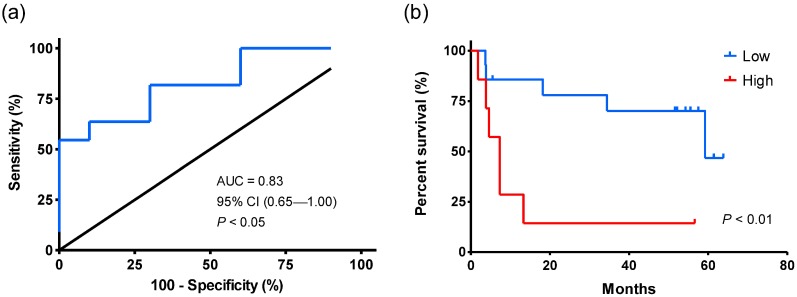
** Kaplan-Meier and ROC analysis for the overall survival of patients from the discovery set (GPL571).** (a) ROC curve shows the sensitivity and specificity of the four-lncRNA signature (HCCLnc-4) in prediction of the patient overall survival, AUC = 0.83 (95% CI: 0.65-1.00, *P* < 0.05). (b) Kaplan-Meier survival curve shows the correlation between HCCLnc-4 and the overall survival of patients. A two-sided log-rank test was performed to evaluate the survival differences between the two curves. The cutoff point was the value that produced the shortest distance to the point of perfect prediction in the ROC curve. The statistical significance level was 0.05.

Univariate Cox regression analysis shows that the levels of HCCLnc-4 were significantly associated with patient survival (HR: 11.697, 95% CI: 2.257-60.618, *P* < 0.01, see in Table 3). As shown in Figure 2a, the AUC value by ROC analysis was 0.83 (95% CI: 0.65-1.00,* P* < 0.05), indicating HCCLnc-4 had high sensitivity and specificity to predict the prognostic survival of patients in the discovery set. And the risk score value of -8.77, which produced the shortest distance to the point of perfect prediction of the ROC curve, was selected as the cutoff point. Using the same cutoff point produced from the ROC curve, patients were divided into low-risk (n = 16) and high-risk groups (n = 6). Kaplan-Meier analysis and log-rank test were then performed. Compared with the low-risk group, patients in the high-risk group had significantly shorter OS time (log-rank test *P* < 0.01, see in Figure 2b).

### Validation of HCCLnc-4 in additional independent test cohorts

**Figure 3:**
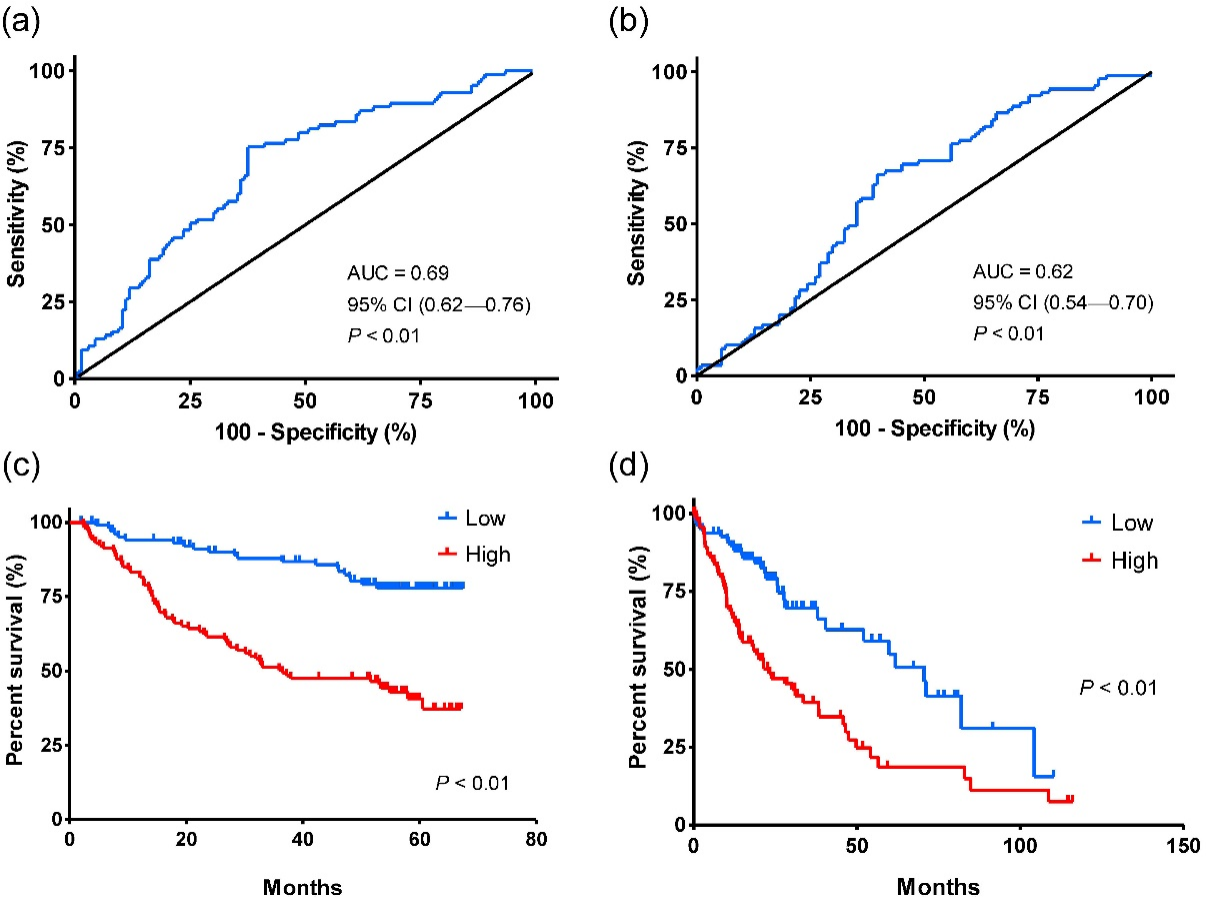
** ROC and Kaplan-Meier analysis for the overall survival of patients in the validation sets.** The patients were divided into low-risk and high-risk groups using the cutoff points that were determined by the ROC curves. (a) ROC curve shows the sensitivity and specificity of the four-lncRNA signature (HCCLnc-4) in prediction of the overall survival of patients from test cohort-1 (GPL3921), AUC = 0.69 (95% CI: 0.62-0.76, *P* < 0.01). (b) ROC curve shows the sensitivity and specificity of HCCLnc-4 in the prediction of the overall survival of patients from test cohort-2 (TANRIC), AUC = 0.62 (95% CI: 0.54-0.70, *P* < 0.01). (c) Kaplan-Meier analysis shows the correlation between HCCLnc-4 and the overall survival of patients from test cohort-1 (GPL3921). A two-sided log-rank test was performed to evaluate the survival differences between the two curves. (d) Kaplan-Meier analysis shows the correlation between HCCLnc-4 and the overall survival of patients from test cohort-2 (TANRIC). A two-sided log-rank test was performed to evaluate the survival differences between the two curves. The statistical significance level was 0.05.

Next, the robustness of HCCLnc-4 was tested in the other two validation cohorts. Univariate Cox regression analysis shows that the levels of the HCCLnc-4 were significantly associated with patient survival both in test cohort-1 (HR: 3.711, 95% CI: 2.263-6.086, *P* < 0.01, see in Table 3), and in test cohort-2 (HR: 2.46, 95%CI: 1.580-3.831, *P* < 0.01, see in Table 3). The area under ROC curves was 0.69 (95% CI: 0.62-0.76, *P* < 0.01) and 0.62 (95% CI: 0.54-0.70, *P* < 0.01) for test cohort-1 and test cohort-2, respectively (Figure 3a and Figure 3b). In addition, Kaplan-Meier analysis and log-rank test show that compared with the low-risk group, patients in the high-risk group had significantly shorter OS time in the two validation cohorts, respectively (in both cohorts, log-rank test *P* < 0.01), as seen in Figure 3c and Figure 3d.

### Independence of HCCLnc-4 for prediction of patient OS from clinicopathological factors

**Figure 4:**
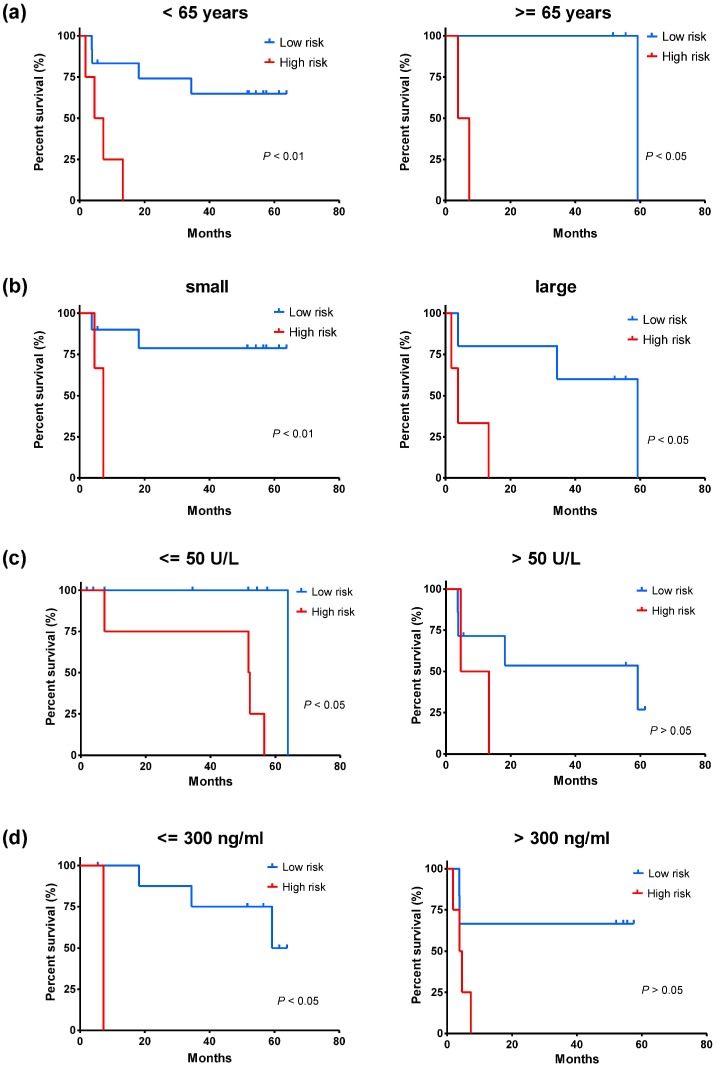
Kaplan-Meier estimates of the overall survival of patients from the discovery set (GPL571) with different ages, tumor size, ALT levels, and AFP levels. The patients were divided into low-risk and high-risk groups using the same cutoff value that was determined by ROC curve of the whole group. (a) Kaplan-Meier analysis for patients of different ages. (b) Kaplan-Meier analysis for patients with different tumor size, tumor <= 5cm, small, and tumor > 5cm, large. (c) Kaplan-Meier analysis for patients with different ALT levels. (d) Kaplan-Meier analysis for patients with different AFP levels. A two-sided log-rank test was performed to evaluate the survival differences between the two curves. The statistical significance level was 0.05.

To distinguish whether HCCLnc-4 could serve as a predictor independent of other clinicopathological parameters, we conducted Kaplan-Meier analysis and log-rank test after stratification by other factors, using the same cutoff value that was determined by ROC curve of the whole group, and multivariate Cox regression analysis. For the discovery cohort, after stratification by age, Kaplan-Meier analysis and log-rank test show that the level of HCCLnc-4 has significant association with OS both in patients < 65 years old (log-rank test *P* < 0.01, see Figure 4a), and in patients >= 65 years old (log-rank test *P* < 0.05, see Figure 4a). After stratification by tumor size, Kaplan-Meier analysis and log-rank test show that the level of HCCLnc-4 has significant association with OS both in patients with small tumors (<= 5 cm) (log-rank test *P* < 0.01, see Figure 4b) and in patients with large tumors (> 5 cm) (log-rank test *P* < 0.05, see Figure 4b). After stratification by alanine aminotransferase (ALT) levels, Kaplan-Meier analysis and log-rank test show that the levels of HCCLnc-4 have a significant association with OS in patients with ALT levels <= 50 U/L (log-rank test *P* < 0.05, see Figure 4c), however has no significant association with OS in patients with ALT levels > 50 U/L (log-rank test *P* > 0.05, see Figure 4c). Similarly, after stratification by AFP levels, Kaplan-Meier analysis and log-rank test show that the levels of HCCLnc-4 have a significant association with OS in patients with AFP levels <= 300 ng/ml (log-rank test *P* < 0.05, see Figure 4d), however has no significant association with OS in patients with AFP levels > 300 ng/ml (log-rank test *P* > 0.05, see Figure 4d).

For test cohort-1, after stratification by other clinicopathological factors, Kaplan-Meier analysis and log-rank test show that the levels of HCCLnc-4 has significant association with OS in patients < 65 years old (log-rank test *P* < 0.01, see Figure S1a), with small tumors (log-rank test *P* < 0.01, see Figure S1b), with large tumors (log-rank test *P* < 0.01, see Figure S1b), male (log-rank test *P* < 0.01, see Figure S1c), with TNM staging I-II (log-rank test *P* < 0.01, see Figure S1d), and with TNM staging III-IV (log-rank test *P* < 0.01, see Figure S1d), respectively, however has no significant association with OS in patients >= 65 years old (log-rank test *P* > 0.05, see Figure S1a), and female (log-rank test *P* > 0.05, see Figure S1c), respectively. In addition, after stratification by more clinicopathological factors, Kaplan-Meier analysis and log-rank test show that the levels of HCCLnc-4 has significant association with OS in patients with AFP levels <= 300 ng/ml (log-rank test *P* < 0.01, see Figure S2a), with AFP levels > 300 ng/ml (log-rank test *P* < 0.01, see Figure S2a), with ALT levels <= 50 U/L (log-rank test *P* < 0.01, see Figure S2b), with ALT levels > 50 U/L (log-rank test *P* < 0.01, see Figure S2b), with cirrhosis (log-rank test *P* < 0.01, see Figure S2c), with chronic carrier status of Hepatitis B Virus (HBV) infection (log-rank test *P* < 0.01, see Figure S2d), and with active viral replication chronic carrier status of HBV infection (log-rank test *P* < 0.01, see Figure S2d), respectively, however has no significant association with OS in patients without cirrhosis (log-rank test *P* > 0.05, see Figure S2c), and normal status of HBV infection (log-rank test *P* > 0.05, see Figure S2d), respectively. Similarly, after stratification by different Barcelona Clinic Liver Cancer (BCLC) staging, and Cancer of The Liver Italian Program (CLIP) staging, Kaplan-Meier analysis and log-rank test show that the levels of HCCLnc-4 has significant association with OS in patients with BCLC staging (0-A) (log-rank test *P* < 0.01, see Figure S3a), with BCLC staging (B-C) (log-rank test *P* < 0.05, see Figure S3a), and with CLIP staging (0-2) (log-rank test *P* < 0.01, see Figure S3b), respectively, however has no significant association with OS in patients with CLIP staging (> 2) (log-rank test P > 0.05, see Figure S3b).

For test cohort-2, after stratification by other clinicopathological factors, Kaplan-Meier analysis and log-rank test show that the levels of HCCLnc-4 has significant association with OS in patients < 65 years old (log-rank test *P* < 0.01, see Figure S4a), >= 65 years old (log-rank test *P* < 0.05, see Figure S4a), male (log-rank test *P* < 0.01, see Figure S4b), with TNM staging I-II (log-rank test *P* < 0.01, see Figure S4c), with TNM staging III-IV (log-rank test *P* < 0.05, see Figure S4c), of American Indian, Alaska Native, and Asian (log-rank test *P* < 0.01, see Figure S4d), and in white patients (log-rank test *P* < 0.05, see Figure S4d), respectively, however has no significant association with OS in female patients (log-rank test *P* > 0.05, see Figure S4b), and in black or African American patients (log-rank test *P* > 0.05, see Figure S4d).

Additionally, multivariate Cox regression analysis suggests that after adjusted by other clinicopathological factors, the levels of HCCLnc-4 was significantly related to patient survival in all the three cohorts (discovery cohort, HR: 36.227, 95% CI: 3.807-344.704, *P* < 0.01; test cohort-1, HR: 3.352, 95% CI: 2.011-5.590, *P* < 0.01, and test cohort-2, HR: 2.243, 95% CI: 1.356-3.711, *P* < 0.01, see Table 3). All the results above indicate that HCCLnc-4 could predict the survival of HCC patients well independent of other clinical performances.

### Comparison of HCCLnc-4 and clinicopathological factors

To determine the superiority of HCCLnc-4 for prediction of HCC patient OS, we calculated AUC that produced by different clinicopathological factors to compare them with that produced by HCCLnc-4. As can be seen in Table 4, AUC produced by HCCLnc-4 was the largest in the discovery set (AUC: 0.773, 95% CI: 0.564-0.981, *P* < 0.05) comparing with those produced by other clinicopathological factors. In addition, AUC produced by HCCLnc-4 was the largest in test cohort-1 (AUC: 0.689, 95% CI: 0.617-0.761, *P* < 0.01), comparing to those produced by TNM staging (AUC: 0.618, 95% CI: 0.539-0.697, *P* < 0.01) and BCLC staging (AUC: 0.630, 95% CI: 0.551-0.708, *P* < 0.01), and was similar to that produced by TNM staging (TNM staging I-II vs III-IV, AUC: 0.639, 95% CI: 0.557-0.721, *P* < 0.01) in test cohort-2 (HCCLnc-4 low vs high, AUC: 0.633, 95% CI: 0.556-0.711, *P* < 0.01). These data suggest that HCCLnc-4 could compete sufficiently with classical clinical and pathological staging systems to predict HCC patient OS.

### Functional prediction of HCCLnc-4

To explore the function of HCCLnc-4, we identified highly positively or negatively correlated PCGs with at least one of 4 lncRNAs by calculating the Pearson correlation coefficient (*P* < 1E-10 as selection criteria) of paired lncRNA and PCGs expression profiles. The functional enrichment analysis of Gene Ontology (GO) and Kyoto Encyclopedia of Genes and Genomes (KEGG) pathway revealed that PCGs positively correlated with lncRNAs were involved in rRNA processing process and spliceosome pathway, while PCGs negatively correlated with lncRNAs were enriched in GO terms mostly related to metabolic process and KEGG pathways, including Complement and coagulation cascades, Metabolic pathways, and PPAR signaling pathway, etc. The HCCLnc-4 associated biological processes and pathways can be found in Table S2.

**Figure 5:**
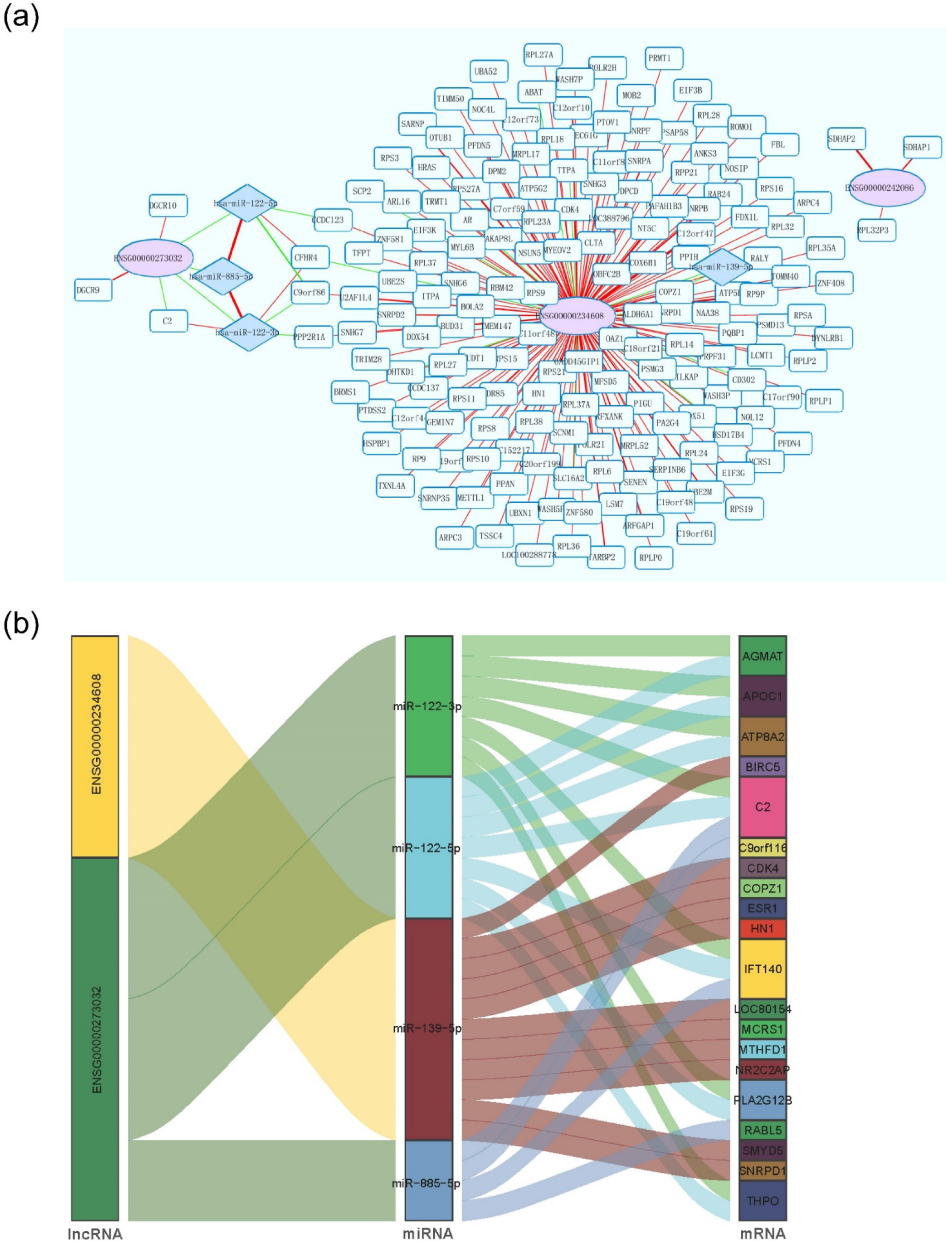
** Interaction network of lncRNA, miRNA, and mRNA in HCC.** (a) Each Ellipse (red) indicates lncRNA associated with the patient overall survival. Each rhombus rectangle indicates miRNA related to lncRNA. The edge color-coded green or red indicates the negative or positive correlation, respectively. The edge size is proportional to the significance of the correlation. (b) Sankey diagram for the ceRNA network in HCC. Each rectangle represents a gene, and the connection degree of each gene is visualized based on the size of the rectangle.

**Figure 6:**
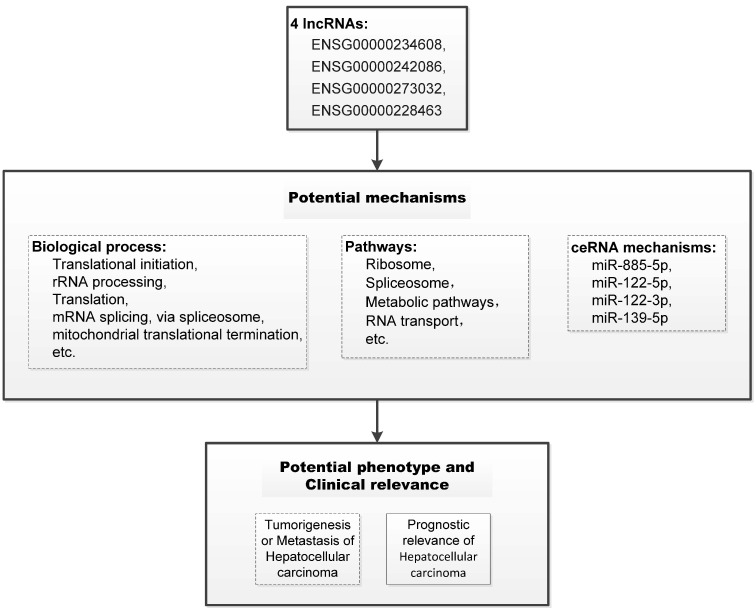
** Schematic of the potential mechanism of the four lncRNAs in the prognostic model of hepatocellular carcinoma.** The potential mechanisms of the four lncRNAs were summarized based on the results of the functional prediction of bioinformatics, statistical analysis of clinical relevance, and previous publications. The dotted boxes, such as biological process, pathways, ceRNA mechanisms, and the potential phenotype of oncobiology, indicate potential biological mechanisms that need to be further revealed. And the solid-line boxes, such as the associations between the four lncRNAs and prognostic relevance of hepatocellular carcinoma, indicate the evidence established in our study.

Using *P* < 1E-20 as selection criteria, 4 miRNAs and 176 mRNAs were identified to be significantly associated with HCCLnc-4. All these four miRNAs (miR-885-5p, miR-122-5p, miR-122-3p, and miR-139-5p) were found to be involved in liver pathology or HCC 24-26. The network revealed that lncRNA especially ENSG00000273032 may intervene in HCC pathogenesis by competitively associated with miR-122 and miR-885, acting as competing endogenous RNAs (ceRNAs), as seen in Figure 5a and Figure 5b. And we summarized our main findings in Figure 6.

## Discussion

The ability to recognize patients with high risk would aid the decision for HCC management. Several clinical prognostic indicators have been proposed to discriminate patients who stand to benefit from liver transplantation. However, they are still insufficient or not sensitive enough for predicting HCC patients at high risk for recurrence and selecting those at low risk 27, 28. Highly sensitive and accurate molecular prognostic biomarkers are sorely needed 29.

As the newly identified class of ncRNA, lncRNAs participate in tumor proliferation, metastasis, invasion, energy regulation, and tumor-initiating cells self-renewal 30. lncRNA based prognostic indexes have been proved to be useful to predict survival, metastasis, and recurrence of tumor patients 31-33. And established by different methods, mRNA 34, lncRNA 35, 36, miRNA 37, or mixed molecule type signature 38 had shown the prognostic value of HCC. For instance, using six prediction machine-learning algorithms, Yuan and colleagues established a metastasis-related signature comprised of five mRNAs and one lncRNA, which presented the well prognostic value of HCC 38. In the present study, using the risk score method and directly based upon different prognostic status, we got an HCC prognosis related four-lncRNA signature and explored the potential implication of lncRNA biomarkers by functional analysis.

Recent studies found that some of the microarray probes on the commonly used arrays are likely to map to lncRNAs 39, 40, which represents a cost-effective way to obtain lncRNA expression profiles. In the present study, by repurposing the expression profiles with OS information of HCC patients from GEO database and TCGA dataset, we obtained lncRNA expression data among the patients, and by Cox regression analysis and risk scoring method, we established a four-lncRNA signature (HCCLnc-4) for predicting HCC patient survival in the discovery cohort. ROC analysis and Kaplan-Meier analysis suggested that HCCLnc-4 was significantly correlated with the survival status of HCC patients. The prognostic value of HCCLnc-4 was further validated in the other two validation cohorts. Stratified analysis and multivariate Cox regression analysis indicated that HCCLnc-4 for HCC patient survival prediction was universal among different subgroups and independent of other prognostic factors. And compared to traditional evaluation systems, such as TNM staging, BCLC staging and CLIP staging systems, a similar or even better HCC prognostic value of HCCLnc-4 could be seen in the same data sets in our research. In addition, recently, Li et al. established a three-gene prognostic signature for patients with HCC, which contained *UPB1*, *SOCS2*, and *RTN3* 34. GSE14520 (containing two batches of samples, GPL571 and GPL3921) was also used as a validation cohort in Li et al's study, and in their study, AUC in time-dependent ROC curve was 0.645 for 1-year survival, 0.638 for 2-year survival, 0.618 for 3-year survival, 0.607 for 4-year survival, and 0.622 for 5-year survival, respectively, compared to 0.83 (GPL571, 95% CI: 0.65-1.00, *P* < 0.05) and 0.69 (GPL3921, 95% CI: 0.62-0.76, *P* < 0.01) of HCCLnc-4 in our study, indicating well or possibly higher sensitivity and specificity of HCCLnc-4 to predict the prognostic survival of HCC patients compared to the recently published mRNA-based signature in the same data sets. These results suggested that HCCLnc-4 was an individualized and robust prognostic marker to predict HCC patient survival.

Though more than ten thousand lncRNAs have been discovered in human, functional studies of these lncRNAs are still in its early stages. *MAPKAPK5* antisense RNA 1 (*MAPKAPK5-AS1*, ENSG00000234608), one of 4 HCC prognosis-related lncRNAs, was differentially expressed between HCC tissues and adjacent non-tumor tissues and negatively associated with OS of HCC patients 41. Similarly, the expression level of *MAPKAPK5-AS1* is positively correlated with HCCLnc-4 and negatively associated with the OS of HCC patients in our study. Upregulation of *MAPKAPK5-AS1* in HCC patients with vascular invasion was also observed 41. Through network analysis, *MAPKAPK5-AS1* is co-expressed with the genes involved in ribosome and spliceosome pathways, which have been partly elucidated elsewhere 42, 43. In addition, another lncRNA, DiGeorge syndrome critical region gene 9 (*DGCR9*, ENSG00000273032), one of the 4 HCC prognosis-related lncRNAs in our study, is also one lncRNA of a 9-lncRNA risk score system for the prognostic prediction of patients with HBV-positive HCC 44. In the study conducted by Dr. Sun and colleagues 44, using one RNA-sequencing dataset from TCGA and three datasets from GEO, they built a 9-lncRNA risk score system, and the expression level of *DGCR9* was negatively associated with the 9-lncRNA risk score and positively correlated with OS of HBV-positive HCC patients. In our study, a similar association between the expression level of *DGCR9* and HCC patient OS was also observed, though our study included patients with HBV either positive or negative. Furthermore, in the network analysis (Figure 5a and Figure 5b), we showed that *DGCR9* (ENSG00000273032) might act as a ceRNA to intervene in HCC pathogenesis by competitively associated to miR-122 and miR-885, which have been found to be involved in liver diseases or liver cancer 45-47. All these results call for further studies to conclude a causal association between these relationships, which may contribute to novel targeted therapy.

Several limitations should be noted in our study. Firstly, we obtain lncRNA expression profiles from the commonly used arrays, so not expression profiles of all lncRNAs were analyzed in our study. Secondly, our model could not include all the potential important prognostic factors, such as steatosis and nonalcoholic fatty liver disease (NAFLD), because the information of these factors was unavailable in the publicly published datasets. Thirdly, prospective studies with larger sample sizes are needed to validate the prognostic value of HCCLnc-4 in HCC patients. Fourthly, the potential biological mechanisms behind the associations between HCCLnc-4 and OS of HCC patients remains to be investigated in further studies.

In conclusion, our study suggested the potential of lncRNA signature as novel candidate biomarkers in the prognosis of HCC, or potential biological functions of lncRNAs in hepatocarcinogenesis.

## Supplementary Material

Supplementary figures and tables.

## Figures and Tables

**Table 1:** Demographics and clinical characteristics of patients in the three sets. (n=447).

	Discovery Set (n=22)	Test Cohort-1 (n=225)	Test Cohort-2 (n=200)
Gender, n (%)			
Female	1 (4.8)	30 (13.6)	70 (35)
Male	20 (95.2)	191 (86.4)	130 (65)
Age, n (%)			
< 65 years	16 (76.2)	196 (88.7)	107 (54)
>= 65 years	5 (23.8)	25 (11.3)	91 (46)
Race, n (%)			
American Indian, Alaska Native, and Asian			55 (28.6)
Black or African American			14 (7.3)
White			123 (64.1)
HBV status1, n (%)			
N		6 (2.8)	
CC		156 (71.6)	
AVR-CC		56 (25.7)	
ALT, n (%)			
low (<= 50 U/L)	12 (57.1)	130 (58.8)	
high (> 50 U/L)	9 (42.9)	91 (41.2)	
AFP, n (%)			
low (<= 300 ng/ml)	10 (50)	118 (54.1)	
high (> 300 ng/ml)	10 (50)	100 (45.9)	
Cirrhosis, n (%)			
No	1 (4.8)	18 (8.1)	
Yes	20 (95.2)	203 (91.9)	
TNM staging, n (%)			
I		93 (41.3)	76 (38)
II		77 (34.2)	48 (24)
III		49 (21.8)	57 (28.5)
IV			5 (2.5)
BCLC staging, n (%)			
0		20 (8.9)	
A		148 (65.8)	
B		22 (9.8)	
C		29 (12.9)	
CLIP staging, n (%)			
0		97 (43.1)	
1		74 (32.9)	
2		35 (15.6)	
3		9 (4.0)	
4		3 (1.3)	
5		1 (0.4)	
Main Tumor Size, n (%)			
Small (<= 5 cm )	13 (61.9)	140 (63.6)	
Large (> 5 cm)	8 (38.1)	80 (36.4)	
Vital Status, n (%)			
Survival	10 (47.6)	136 (61.5)	111 (55.5)
Death	11 (52.4)	85 (38.5)	89 (44.5)
Overall survival time (months)			
Mean	31.8	40.4	24.6
Range	1.8-63.8	2-67.4	0-115.9

**^1^**N, Normal, CC, chronic carrier, and AVR-CC, active viral replication chronic carrier. Abbreviations: HBV, hepatitis B virus; ALT, alanine aminotransferase; AFP, alpha-fetoprotein; TNM, TNM (tumor, lymph node, and metastasis) Classification of Malignant Tumors; BCLC, Barcelona Clinic Liver Cancer; CLIP, Cancer of The Liver Italian Program.

**Table 2:** Basic information of four lncRNAs in the four-lncRNA signature to predict the overall survival time of HCC patients.

Ensembl ID	Probe ID	Name	Chromosome
ENSG00000234608	64432_at	MAPKAPK5 antisense RNA 1 [Source: HGNC Symbol;Acc:HGNC:24091]	Chr 12: 111839764 - 111842902 (-1)
ENSG00000242086	222021_x_at	long intergenic non-protein coding RNA 969 [Source: HGNC Symbol;Acc:HGNC:48729]	Chr 3: 195658062 - 1957399642 (1)
ENSG00000273032	215003_at	DiGeorge syndrome critical region gene 9 (non-protein coding) [Source: HGNC Symbol;Acc:HGNC:17227], also known as DGS-A	Chr 22: 19017834 - 19020248 (1)
ENSG00000228463	221634_at	AP006222.1 (Ribosomal Protein L23a pseudogene)	Chr 1: 257864 - 297502 (-1)

**Table 3:** Multivariate Cox regression analysis of overall survival time in each set.

		Univariate model			Multivariate model		
		HR	95% CI of HR	*P*-Value	HR	95% CI of HR	*P*-Value
**Discovery set (n=22)**							
Age (years)	< 65	1 (reference)			1 (reference)		
	>= 65	1.063	0.281-4.015	> 0.05	0.226	0.038-1.341	> 0.05
Main tumor size (cm)	small (<= 5)	1 (reference)			1 (reference)		
	large (> 5)	2.24	0.682-7.354	> 0.05	8.487	1.093-65.897	< 0.05
ALT (U/L)	low (<= 50)	1 (reference)			1 (reference)		
	high (> 50)	1.802	0.538-6.032	> 0.05	3.358	0.711-15.857	> 0.05
AFP (ng/ml)	low (<= 300)	1 (reference)			1 (reference)		
	high (> 300)	2.976	0.738-11.998	> 0.05	1.441	0.279-7.432	> 0.05
HCCLnc-4^1^	low	1 (reference)			1 (reference)		
	high	11.697	2.257-60.618	< 0.01	36.227	3.807-344.704	< 0.01
**Test cohort-1 (n=225)**							
Age (years)	< 65	1 (reference)			1 (reference)		
	>= 65	0.542	0.236-1.244	> 0.05	0.665	0.255-1.735	> 0.05
Main tumor size (cm)	small (<= 5)	1 (reference)			1 (reference)		
	large (> 5)	1.924	1.252-2.956	< 0.01	1.011	0.551-1.855	> 0.05
Gender	female	1 (reference)			1 (reference)		
	male	1.7	0.82-3.521	> 0.05	1.512	0.707-3.236	> 0.05
TNM staging	I-II	1 (reference)			1 (reference)		
	III-IV	3.513	2.24-5.511	< 0.01	1.602	0.725-3.541	> 0.05
HBV status^2^	N	1 (reference)			1 (reference)		
	CC	1.062	0.259-4.353	> 0.05	1.075	0.254-4.540	> 0.05
	AVR-CC	1.42	0.336-6	> 0.05	1.797	0.406-7.948	> 0.05
ALT (U/L)	low (<= 50)	1 (reference)			1 (reference)		
	high (> 50)	1.08	0.703-1.658	> 0.05	0.805	0.507-1.280	> 0.05
AFP (ng/ml)	low (<= 300)	1 (reference)			1 (reference)		
	high (> 300)	1.629	1.063-2.497	< 0.05	1.139	0.710-1.825	> 0.05
BCLC staging	0-A	1 (reference)			1 (reference)		
	B-C	3.546	2.27-5.54	< 0.01	1.073	0.524-2.197	> 0.05
Cirrhosis	No	1 (reference)			1 (reference)		
	Yes	4.622	1.137-18.797	< 0.05	4.363	1.054-18.064	< 0.05
CLIP staging	0-2	1 (reference)			1 (reference)		
	>2	3.736	1.974-7.071	< 0.01	1.902	0.861-4.203	> 0.05
HCCLnc-4^1^	low	1 (reference)			1 (reference)		
	high	3.711	2.263-6.086	< 0.01	3.352	2.011-5.590	< 0.01
**Test cohort-2 (n=200)**							
Age (years)	< 65	1 (reference)			1 (reference)		
	>= 65	0.834	0.545-1.277	> 0.05	0.834	0.508-1.369	> 0.05
Gender	female	1 (reference)			1 (reference)		
	male	1.257	0.813-1.943	> 0.05	0.992	0.586-1.678	> 0.05
TNM staging	I-II	1 (reference)			1 (reference)		
	III-IV	2.172	1.392-3.391	< 0.01	2.139	1.296-3.531	< 0.01
Race	American Indian, Alaska Native, and Asian	1 (reference)			1 (reference)		
	Black or African American	0.848	0.347-2.075	> 0.05	1.253	0.45-3.489	> 0.05
	White	0.512	0.312-0.841	< 0.01	0.476	0.267-0.848	< 0.05
HCCLnc-4^1^	low	1 (reference)			1 (reference)		
	high	2.46	1.58-3.831	< 0.01	2.243	1.356-3.711	< 0.01

^1^Patients were divided into low-risk and high-risk groups by the cutoff point that produced the shortest distance to the point of perfect prediction of the ROC curve. ^2^N, Normal, CC, chronic carrier, and AVR-CC, active viral replication chronic carrier. Abbreviations: BCLC, Barcelona Clinic Liver Cancer; CLIP, Cancer of with Liver Italian Program; HR, hazard ratio; CI, confidence interval; HCCLnc-4, four-lncRNA signature for prediction of hepatocellular carcinoma patients; ALT, alanine aminotransferase; AFP, alpha-fetoprotein; TNM, TNM (tumor, lymph node, and metastasis) Classification of Malignant Tumours; HBV, hepatitis B virus.

**Table 4:** ROC analysis of clinical and pathological parameters and the four-lncRNA signature in the three datasets.

	Discovery Set (n=22)			Test Cohort-1 (n=225)			Test Cohort-2 (n=200)		
	AUC	95% CI of AUC	*P*-Value	AUC	95% CI of AUC	*P*-Value	AUC	95% CI of AUC	*P*-Value
**Gender (female / male)**				0.534	0.457-0.611	0.398	0.461	0.380-0.542	0.344
**Age (< 65 years / >= 65 years )**	0.536	0.284-0.788	0.778	0.465	0.388-0.543	0.388	0.521	0.439-0.602	0.618
**Race (white / others^1^)**	0.673	0.436-0.909	0.181				0.482	0.402-0.563	0.67
**HBV status (normal / active)**				0.503	0.424-0.582	0.94			
**ALT (<= 50 U/L / > 50 U/L)**	0.623	0.378-0.868	0.342	0.519	0.441-0.598	0.633			
**AFP (<= 300ng/ml / > 300 ng/ml)**	0.6	0.346-0.854	0.45	0.568	0.489-0.646	0.093			
**Cirrhosis (yes/no)**				0.547	0.47-0.624	0.239			
**TNM staging (I-II / III-IV)**				0.618	0.539-0.697	< 0.01	0.639	0.557-0.721	< 0.01
**BCLC staging (0-A / B-C)**				0.630	0.551-0.708	< 0.01			
**CLIP staging (0-2 / > 2)**				0.550	0.470-0.630	0.211			
**Main Tumor Size (<= 5 cm / > 5 cm)**				0.568	0.490-0.646	0.09			
**HCCLnc-4^2^ (low / high)**	0.773	0.564-0.981	< 0.05	0.689	0.617-0.761	< 0.01	0.633	0.556-0.711	< 0.01

^1^Containing American Indian, Alaska Native, Asian, and black or African American. ^2^Patients were divided into low-risk and high-risk groups by the cutoff point that produced the shortest distance to the point of perfect prediction of the ROC curve. Abbreviations: BCLC, Barcelona Clinic Liver Cancer; CLIP, Cancer of The Liver Italian Program; HCCLnc-4, four-lncRNA signature for prediction of hepatocellular carcinoma patients; ALT, alanine aminotransferase; AFP, alpha-fetoprotein; TNM, TNM (tumor, lymph node, and metastasis) Classification of Malignant Tumours; HBV, hepatitis B virus.

## Abbreviations

HCC: hepatocellular carcinoma
lncRNA: long non-coding RNA
GEO: Gene Expression Omnibus
TCGA: The Cancer Genome Atlas
AUC: area under curve
AFP: alpha-fetoprotein
ncRNA: non-coding RNA
OS: overall survival
ALT: alanine aminotransferase
HBV: Hepatitis B Virus
BCLC: Barcelona Clinic Liver Cancer
CLIP: Cancer of The Liver Italian Program
GO: Gene Ontology
KEGG: Kyoto Encyclopedia of Genes and Genomes
ceRNA: competing endogenous RNAs
