# Supplementary material for: The Prediction of Survival in Hepatocellular Carcinoma Based on A Four Long Non-coding RNAs Expression Signature
Source: J Cancer. 2020 Apr 12;11(14):4132–44. doi: 10.7150/jca.40621 (PMC7196252; doi:10.7150/jca.40621)
Supplement: Supplementary file 1 — Supplementary figures and tables. [file jcav11p4132s1.pdf]

The Prediction of Survival in Hepatocellular Carcinoma Based on A Four Long Non-coding RNAs Expression Signature

Zongxing Yang [1,*], Yuhan Yang [2,*], Gang Zhou [2], Yan Luo [2], Wenjun Yang [2], Youliang Zhou [2], Jin Yang [2,#]

[1] The Second Department of Infectious Disease, Xixi Hospital of Hangzhou, the Affiliated Hospital of Zhejiang Chinese Medical University, Hangzhou, Zhejiang 310023, P.R. China

[2] Center for Translational Medicine, the affiliated hospital of Hangzhou Normal University, Hangzhou Normal University, Hangzhou, Zhejiang 310015, P.R. China

* These authors contributed equally to this work.

[#] Address correspondence to Jin Yang, Ph.D., Department of Translational Medicine Center, Affiliated Hospital of Hangzhou Normal University, Hangzhou Normal University, Hangzhou, Zhejiang 310015, P.R. China. Tel: 086-571-88358065. Fax: 086-571-88358065. E-mail: hz_zhiy@163.com.

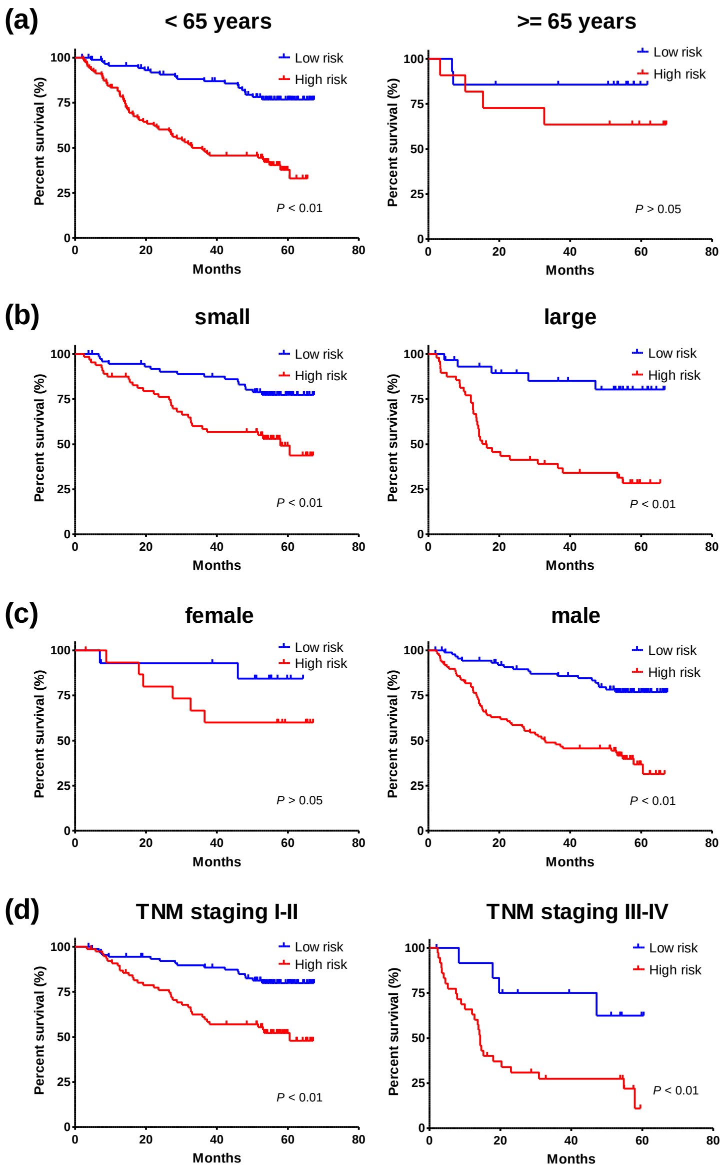

Figure S1. Kaplan–Meier estimates of the overall survival of patients from test cohort-1 (GPL3921) with different ages, tumor size, gender, and TNM stages. The patients were divided into low-risk and high-risk groups using the same cutoff value that was determined by ROC curve of the whole group. (a) Kaplan–Meier analysis for patients with different ages. (b) Kaplan–Meier analysis for patients with different tumor size, tumor <= 5cm, small, and tumor > 5cm, large. (c) Kaplan–Meier analysis for patients in different sex groups. (d) Kaplan–Meier analysis for patients with different TNM stages. The survival differences between the two curves were determined by two-sided log-rank test. The statistical significance level was 0.05.

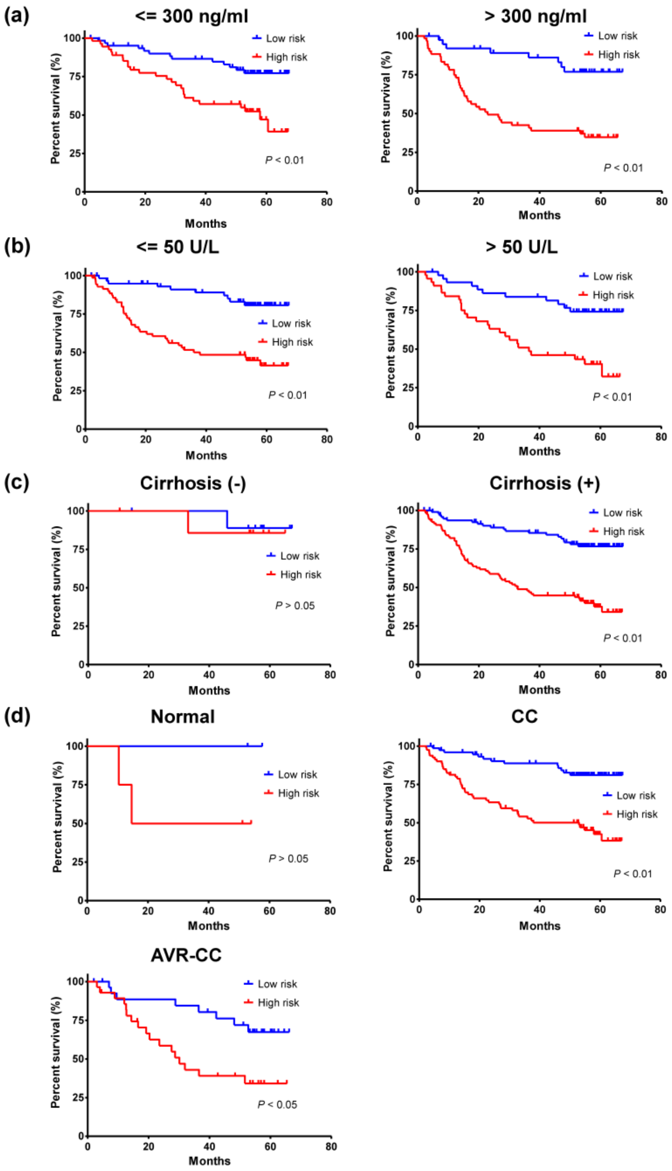

Figure S2. Kaplan–Meier estimates of the overall survival of patients from test cohort-1 (GPL3921) with different AFP levels, ALT levels, hepatic cirrhosis status, and HBV infection status. The patients were divided into low-risk and high-risk groups using the same cutoff value that was determined by ROC curve of the whole group. (a) Kaplan–Meier analysis for patients with different AFP levels. (b) Kaplan–Meier analysis for patients with different ALT levels. (c) Kaplan–Meier analysis for patients with different hepatic cirrhosis status. (d) Kaplan–Meier analysis for patients with different HBV infection status. CC, chronic carrier, and AVR-CC, active viral replication chronic carrier. The survival differences between the two curves were determined by two-sided log-rank test. The statistical significance level was 0.05.

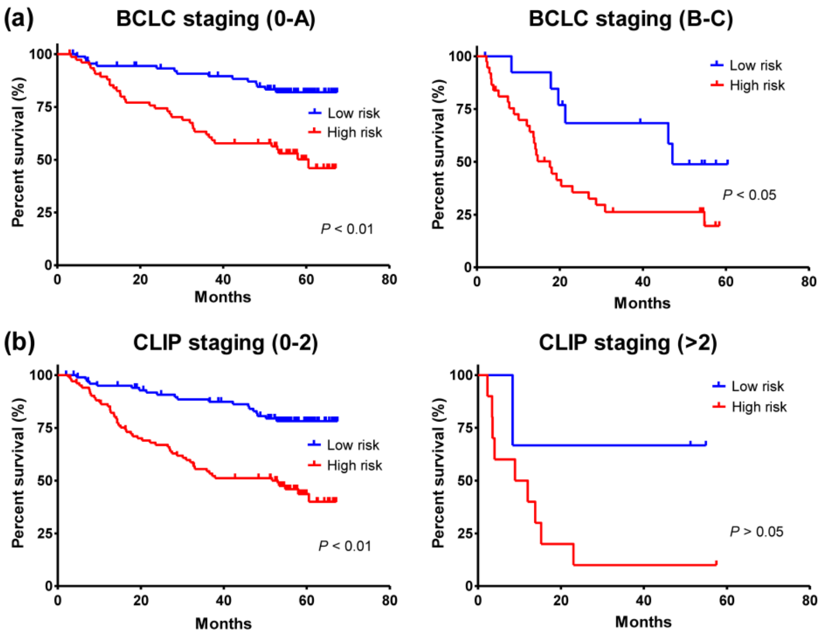

Figure S3. Kaplan–Meier estimates of the overall survival of patients from test cohort-1 (GPL3921) with different BCLC stages, and CLIP stages. The patients were divided into low-risk and high-risk groups using the same cutoff value that was determined by ROC curve of the whole group. (a) Kaplan–Meier analysis for patients with different BCLC stages. (b) Kaplan–Meier analysis for patients with different CLIP stages. The survival differences between the two curves were determined by two-sided log-rank test. The statistical significance level was 0.05.

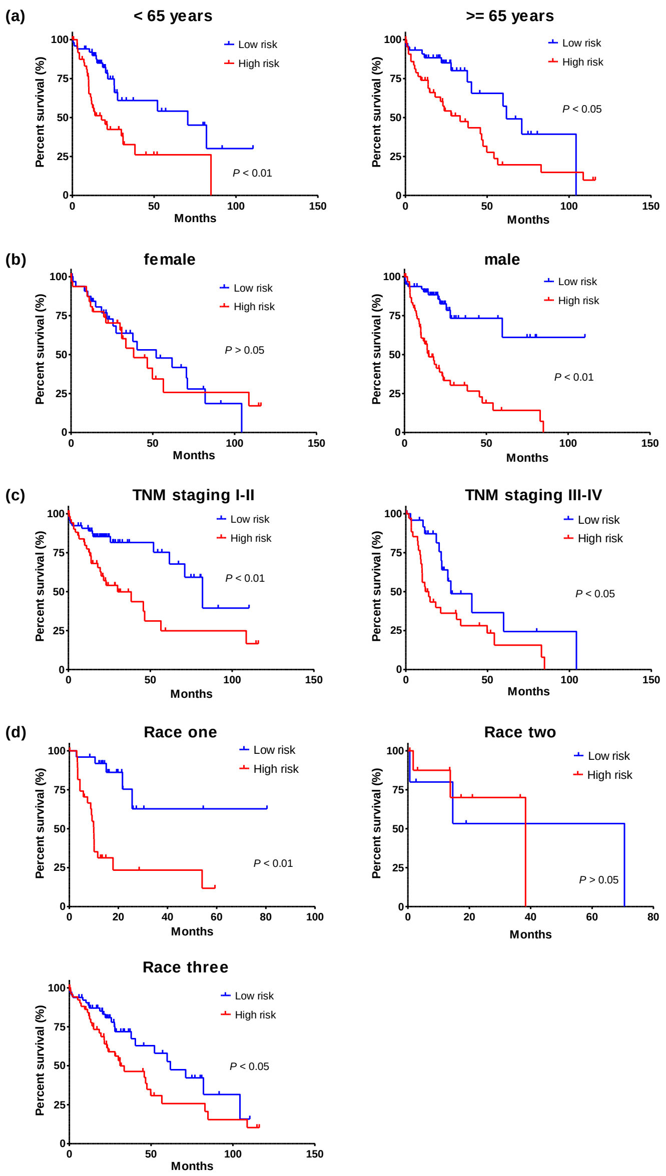

Figure S4. Kaplan–Meier estimates of the overall survival of patients from test cohort-2 (TANRIC) with different ages, gender, TNM stages, and races. The patients were divided into low-risk and high-risk groups using the same cutoff value that was determined by ROC curve of the whole group. (a) Kaplan–Meier analysis for patients with different ages. (b) Kaplan–Meier analysis for patients in different sex groups. (c) Kaplan–Meier analysis for patients with different TNM stages. (d) Kaplan–Meier analysis for patients with different races. Race one, American Indian, Alaska Native, and Asian, Race two, black or African American, and Race three, white. The survival differences between the two curves were determined by two-sided log-rank test. The statistical significance level was 0.05.

Table S1. Sample list and corresponding information of patients in the three sets.

ID	Overall survival status[1]	Overall survival time (months)	Age (years)	Gender[2]	Race	HBV status[3]	ALT(<=/> 50 U/L)	AFP(<=/> 300 ng/ml)	Cirrhosis[4]	TNM staging	BCLC staging	CLIP staging	Main Tumor Size(<=/>5 cm)
**GPL571 (discovery cohort)**													
GSM362950	0	57.5	45	M			low	high	Y	I	A	1	small
GSM362952	0	54.3	61	M			low	high	Y	I	A	1	small
GSM363420	1	7.3	72	M			low	high	Y				small
GSM363422	0	51.6	67	M			low	low	Y				small
GSM363424	0	63.8	43	M			low	low	Y				small
GSM363426	1	1.8	33	M			low	high	Y				large
GSM363428	1	3.8	76	M			low	high	Y				large
GSM363432	1	34.4	41	M			low	low	Y				large
GSM363438	0	56.6	55	M			low	low	Y				small
GSM363444	1	7.3	46	M			low	low	Y				small
GSM363446	0	52.1	50	M			low	high	Y				large
GSM363448	0	51.7	60	F			low	low	Y				small
GSM362947	1	59.2	67	M			high	low	Y	IIIA	B	1	large
GSM362948	0	61.4	37	M			high	low	Y	II	A	1	small
GSM362949	1	18.2	37	M			high	low	Y				small
GSM362954	0	5.5	40	M			high	low	Y	I	A	0	small
GSM362956	1	13.3	33	M			high	.	Y	IIIA	B	1	large
GSM363430	1	3.7	47	M			high	high	Y				small
GSM363436	1	3.8	48	M			high	high	Y				large
GSM363440	0	55.5	73	M			high	high	N				large
GSM363442	1	4.6	42	M			high	high	Y				small

GSM363434												
**GPL3921 (test cohort-1)**												
GSM362958	1	28.2	56	M	CC	low	low	Y	II	A	0	large
GSM362964	0	67.4	50	M	CC	high	low	Y	I	A	0	small
GSM362965	0	66.6	57	M	CC	low	low	N	II	A	0	large
GSM362966	0	66.1	58	M	AVR-CC	high	low	Y	I	A	0	small
GSM362970	0	67.3	39	M	CC	low	low	N	I	0	0	small
GSM362972	0	66	45	M	CC	low	low	Y	I	0	0	small
GSM362976	0	67	50	M	CC	high	low	Y	I	A	0	small
GSM362977	0	66.3	41	M	CC	low	low	Y	I	A	0	large
GSM362982	0	64.6	39	M	CC	high	low	Y	I	A	0	small
GSM362984	0	51.4	49	M	CC	low	low	Y	II	A	0	small
GSM362986	0	64.5	55	M	AVR-CC	low	low	Y	II	0	0	small
GSM362987	0	60.4	60	M	AVR-CC	high	low	Y	II	A	0	small
GSM363012	1	4.5	61	M	CC	low	low	Y	I	A	0	large
GSM363014	0	60.7	61	M	CC	low	low	Y	I	A	0	small
GSM363029	0	51.3	48	M	CC	low	low	Y	I	A	0	small
GSM363031	0	52.8	58	M	CC	low	low	Y	II	A	0	small
GSM363034	1	45.9	30	F	CC	low	low	N	I	A	0	small
GSM363035	0	56.1	40	M	CC	high	low	Y	I	A	0	large
GSM363037	0	61.2	54	M	AVR-CC	low	low	Y	I	A	0	small
GSM363038	1	15.1	41	M	CC	low	low	Y	II	A	0	small
GSM363039	0	52.8	54	M	N	low	low	Y	I	A	0	large

GSM363049	0	62.4	45	M	AVR-CC	low	high	Y	I	A	0	.
GSM363053	0	57	57	F	CC	low	low	Y	I	A	0	small
GSM363054	0	53.8	51	M	CC	low	low	Y	II	A	0	large
GSM363072	0	10.4	49	M	CC	low	low	N	I	A	0	small
GSM363074	0	57.7	41	M	CC	high	low	Y	II	A	0	small
GSM363078	1	57.9	47	M	CC	low	low	Y	IIIB	A	0	small
GSM363082	0	57	64	M	CC	low	low	Y	II	0	0	small
GSM363083	0	53.3	45	M	AVR-CC	low	low	N	I	0	0	small
GSM363084	0	62.6	67	M	CC	high	low	Y	I	A	0	large
GSM363102	1	37.9	49	M	CC	low	low	Y	II	A	0	large
GSM363104	1	35.9	59	M	CC	low	low	Y	I	A	0	small
GSM363108	0	48.8	49	M	CC	high	low	Y	II	A	0	large
GSM363109	0	55.2	50	M	CC	low	low	Y	II	A	0	small
GSM363115	0	66.6	74	M	CC	low	low	Y	I	A	0	small
GSM363122	0	55.4	49	M	AVR-CC	high	low	Y	I	A	0	small
GSM363123	0	42.7	48	M	AVR-CC	high	low	Y	II	A	0	large
GSM363124	0	53.5	67	M	CC	low	.	Y	I	A	0	small
GSM363128	1	30.1	36	M	AVR-CC	high	low	Y	II	A	0	small
GSM363130	0	61.8	72	M	CC	low	low	Y	II	A	0	large
GSM363143	1	32.6	48	F	CC	low	low	Y	II	0	0	small
GSM363144	0	51.1	72	M	N	low	low	Y	I	A	0	small
GSM363145	1	33	43	M	CC	high	low	N	I	A	0	small
GSM363149	0	14.4	53	M	CC	low	low	N	I	A	0	large

GSM363170	0	53.3	59	M	CC	low	low	Y	I	0	0	small
GSM363172	1	6.7	67	M	CC	high	low	Y	II	A	0	small
GSM363176	0	50.7	65	F	CC	low	low	Y	I	A	0	small
GSM363178	0	57.3	42	M	CC	low	low	N	II	A	0	small
GSM363182	0	52.7	50	M	CC	low	low	Y	I	A	0	small
GSM363186	1	7.6	45	M	AVR-CC	low	low	Y	II	A	0	small
GSM363188	0	57.3	35	M	AVR-CC	high	low	Y	II	A	0	small
GSM363192	0	52.2	50	M	CC	high	low	Y	I	0	0	small
GSM363196	0	57.7	42	M	CC	high	low	Y	I	A	0	small
GSM363198	0	57.9	41	M	AVR-CC	high	low	Y	I	A	0	small
GSM363202	0	60	33	M	CC	low	low	Y	I	A	0	small
GSM363205	0	58	70	M	CC	low	high	N	I	0	0	small
GSM363213	0	57.6	50	M	N	high	low	N	II	C	0	large
GSM363222	0	48.4	59	M	AVR-CC	high	low	Y	I	A	0	small
GSM363226	0	60.4	54	M	AVR-CC	high	low	Y	IIIC	C	0	large
GSM363230	0	4.8	40	M	CC	high	low	Y	II	A	0	large
GSM363232	0	57.9	49	M	AVR-CC	low	low	N	II	A	0	small
GSM363247	0	52.9	44	M	CC	high	low	N	I	A	0	small
GSM363269	1	52.7	55	M	AVR-CC	low	low	Y	I	A	0	small
GSM363271	0	28.7	52	M	CC	low	low	Y	IIIC	C	0	large
GSM363273	0	58	56	M	CC	high	low	Y	I	A	0	small

GSM363290	0	54.7	63	F	CC	high	low	Y	I	0	0	small
GSM363291	1	50.1	54	M	CC	high	low	Y	I	A	0	small
GSM363292	0	59.9	53	M	CC	high	low	Y	I	A	0	small
GSM363293	0	61.5	54	M	CC	high	low	Y	I	A	0	small
GSM363309	1	53	34	M	CC	low	low	Y	II	0	0	small
GSM363310	1	23.5	41	M	AVR-CC	high	low	Y	II	A	0	small
GSM363311	0	19	77	M	CC	low	low	Y	II	A	0	large
GSM363312	0	55.3	53	F	CC	high	low	Y	II	A	0	small
GSM363313	0	36.6	71	M	CC	low	low	Y	II	A	0	large
GSM363327	0	60.1	52	M	AVR-CC	high	low	Y	I	A	0	small
GSM363328	1	12	63	M	AVR-CC	low	high	Y	I	A	0	large
GSM363329	0	53.2	56	M	AVR-CC	low	low	Y	I	A	0	small
GSM363330	1	12.6	50	M	CC	low	low	Y	I	A	0	large
GSM363335	0	59.4	45	M	CC	low	low	Y	I	A	0	small
GSM363336	1	8	63	M	CC	low	low	Y	I	A	0	small
GSM363344	0	38.7	52	F	CC	low	low	Y	II	A	0	small
GSM363346	1	14.3	51	M	AVR-CC	high	low	Y	IIIB	A	0	large
GSM363348	0	14.3	41	M	CC	high	low	Y	I	A	0	small
GSM363355	1	9.1	53	M	CC	low	low	Y	II	A	0	small
GSM363357	1	8.8	47	F	AVR-CC	low	low	Y	II	A	0	large
GSM363358	0	53.9	56	M	CC	low	low	Y	II	A	0	small
GSM363360	0	53.3	71	M	CC	low	high	Y	I	A	0	small

GSM363362	1	5.8	53	M	CC	low	low	Y	II	A	0	small
GSM363364	1	27.1	49	M	CC	low	low	Y	I	A	0	small
GSM363366	0	52.3	73	M	CC	low	low	Y	I	A	0	large
GSM363371	0	66.3	69	F	CC	high	low	Y	I	A	0	small
GSM363376	0	61.5	62	M	CC	high	low	Y	I	0	0	small
GSM363378	1	31.9	47	M	AVR-CC	low	low	Y	I	A	0	small
GSM363384	1	51.6	43	M	AVR-CC	high	low	Y	II	A	0	small
GSM363388	0	57.3	58	F	CC	high	low	Y	I	0	0	small
GSM363391	0	57.3	55	M	CC	low	low	Y	II	0	0	small
GSM363400	0	55.6	58	M	CC	low	low	Y	II	A	0	small
GSM362959	1	9.5	45	M	AVR-CC	high	high	Y	I	A	1	small
GSM362960	0	66.1	21	M	CC	high	high	Y	II	A	1	small
GSM362978	1	28.8	61	M	AVR-CC	high	low	Y	I	A	1	small
GSM362983	0	64.2	32	M	CC	high	high	Y	I	A	1	small
GSM362988	0	51.1	52	F	AVR-CC	low	high	Y	I	0	1	small
GSM362992	0	64.3	41	F	CC	high	.	Y	I	A	1	large
GSM362993	0	3.8	50	M	CC	low	high	Y	I	A	1	small
GSM362994	0	54.8	49	M	CC	low	high	Y	II	A	1	small
GSM363008	0	58.4	50	F	CC	low	low	Y	II	B	1	small
GSM363009	0	58.9	50	M	CC	high	high	Y	II	A	1	large
GSM363010	0	62	47	M	CC	high	.	Y	I	A	1	large
GSM363013	1	7.7	58	M	CC	high	high	Y	II	A	1	small
GSM363015	0	59.7	39	M	CC	low	high	N	I	A	1	large

GSM363016	0	2	44	M	AVR-CC	low	low	Y	IIIA	B	1	large
GSM363032	0	24.9	52	M	CC	low	high	Y	IIIB	A	1	large
GSM363033	1	36.4	63	M	AVR-CC	low	high	Y	I	A	1	small
GSM363036	1	32.7	65	M	CC	high	low	Y	II	A	1	small
GSM363055	0	7.3	55	F	CC	low	high	Y	I	A	1	large
GSM363057	1	60.5	63	M	CC	high	low	Y	II	A	1	small
GSM363073	1	26.5	41	M	CC	low	high	Y	II	A	1	small
GSM363075	1	18	54	F	CC	low	low	Y	IIIB	C	1	large
GSM363077	0	52.7	40	M	CC	low	high	Y	I	0	1	small
GSM363081	1	5.2	34	M	CC	high	low	Y	IIIA	C	1	large
GSM363087	0	54	40	M	N	low	high	Y	I	A	1	small
GSM363098	0	56.9	60	M	AVR-CC	low	high	Y	II	0	1	small
GSM363101	0	56.3	50	M	CC	low	high	Y	I	A	1	small
GSM363105	0	9.5	36	M	AVR-CC	high	low	Y	II	A	1	small
GSM363107	1	7	71	F	AVR-CC	low	high	Y	I	A	1	small
GSM363125	1	16.5	43	M	AVR-CC	low	high	Y	II	A	1	large
GSM363126	0	59.5	65	M	CC	low	high	Y	IIIB	A	1	large
GSM363127	1	22.2	55	M	CC	low	high	Y	II	A	1	small
GSM363147	1	28.7	44	M	AVR-CC	high	low	Y	II	B	1	small
GSM363150	1	3.3	68	M	CC	low	high	Y	II	A	1	large
GSM363151	0	67.1	70	F	CC	low	low	Y	II	A	1	small

GSM363164	0	55.2	27	F	CC	low	high	N	I	A	1	small
GSM363166	0	53.8	59	M	CC	low	high	Y	IIIB	A	1	large
GSM363168	0	53.8	51	M	CC	low	low	Y	IIIA	B	1	large
GSM363184	0	16.3	34	M	AVR-CC	high	low	Y	IIIA	B	1	large
GSM363190	0	4.8	41	M	AVR-CC	high	low	Y	II	A	1	small
GSM363200	1	23.9	26	M	CC	low	high	Y	II	A	1	small
GSM363207	1	53.3	53	M	CC	low	high	Y	I	A	1	large
GSM363209	0	60.8	45	F	CC	low	high	Y	I	A	1	small
GSM363215	0	18.6	48	M	CC	low	high	Y	I	0	1	small
GSM363217	0	54.5	58	M	CC	low	low	N	IIIC	C	1	small
GSM363224	0	57.9	70	M	CC	low	high	Y	I	A	1	small
GSM363237	0	57.5	69	M	CC	high	high	Y	I	A	1	large
GSM363239	1	42.2	54	M	AVR-CC	high	low	Y	II	A	1	small
GSM363241	0	54.2	55	M	CC	high	high	Y	I	A	1	large
GSM363243	0	55.8	69	M	CC	low	low	Y	I	A	1	small
GSM363249	0	59.2	60	F	CC	low	high	Y	II	A	1	small
GSM363264	1	17.8	43	M	CC	high	low	Y	III	B	1	large
GSM363265	0	65.1	41	M	CC	low	low	Y	II	A	1	small
GSM363266	1	2.5	35	M	CC	high	low	Y	IIIB	C	1	small
GSM363267	1	47.9	54	M	CC	low	low	Y	II	A	1	small
GSM363268	0	56	56	M	AVR-CC	low	high	Y	I	A	1	small
GSM363270	1	21.3	58	M	CC	high	low	Y	II	B	1	small
GSM363275	0	59.8	67	F	CC	high	low	Y	IIIB	A	1	small
GSM363288	0	60.3	56	M	CC	low	high	Y	I	A	1	small

GSM363289	0	54.2	46	M	CC	low	high	Y	I	A	1	small
GSM363297	0	3	58	F	.	low	high	Y	IIIB	A	1	large
GSM363298	0	53.6	52	M	CC	low	high	N	I	A	1	small
GSM363315	0	65.4	43	M	CC	high	high	Y	II	A	1	small
GSM363316	0	54.7	35	F	AVR-CC	low	high	Y	I	A	1	small
GSM363317	0	62.8	58	M	CC	high	high	Y	I	0	1	small
GSM363331	1	36.5	54	F	AVR-CC	high	high	Y	I	A	1	large
GSM363332	1	12.6	54	M	AVR-CC	low	high	Y	I	A	1	large
GSM363333	1	13.5	48	M	CC	low	high	Y	II	A	1	large
GSM363337	0	65.5	53	F	CC	low	high	Y	I	A	1	small
GSM363339	0	65.4	49	F	AVR-CC	high	high	Y	II	0	1	small
GSM363341	0	65.4	32	M	CC	low	high	Y	II	A	1	large
GSM363343	0	65.1	30	F	CC	low	high	N	I	A	1	small
GSM363350	0	62.9	43	M	CC	low	high	Y	I	A	1	large
GSM363352	1	16.2	51	M	CC	high	high	Y	II	A	1	small
GSM363354	1	10.4	66	M	N	low	high	Y	I	A	1	large
GSM362971	1	48.1	52	M	AVR-CC	high	high	Y	I	A	2	small
GSM363011	1	17.6	57	M	CC	low	high	Y	II	B	2	small
GSM363017	1	12.7	50	M	AVR-CC	low	low	Y	IIIB	B	2	large
GSM363030	1	4.5	58	M	CC	high	high	Y	IIIA	A	2	small
GSM363048	1	20.3	50	M	AVR-CC	low	high	Y	IIIA	B	2	large

GSM363050	0	54.2	32	M	AVR-CC	high	high	Y	II	B	2	small
GSM363051	0	20.6	43	M	CC	low	high	Y	IIIA	B	2	large
GSM363052	1	14.3	46	M	CC	high	low	Y	IIIA	C	2	large
GSM363056	1	19.2	59	F	AVR-CC	high	high	Y	II	B	2	small
GSM363069	1	13.6	34	M	CC	high	high	Y	IIIA	C	2	large
GSM363079	1	7.8	53	M	CC	high	high	Y	IIIA	C	2	large
GSM363085	1	54.8	49	M	CC	high	high	Y	IIIA	B	2	large
GSM363086	0	60.5	44	M	AVR-CC	low	high	Y	II	A	2	small
GSM363099	1	30.9	50	M	CC	low	high	Y	III	B	2	large
GSM363106	1	14.6	53	M	N	low	high	Y	IIIA	C	2	large
GSM363142	1	46.1	28	M	CC	high	high	Y	II	B	2	small
GSM363146	0	54.9	50	M	CC	high	low	Y	IIIA	C	2	large
GSM363148	0	52.4	64	M	CC	high	high	Y	II	A	2	small
GSM363169	0	39.4	45	M	AVR-CC	high	high	Y	IIIA	B	2	large
GSM363174	0	67.1	48	M	CC	high	high	Y	II	A	2	small
GSM363180	1	47.1	52	M	CC	low	high	Y	IIIA	B	2	large
GSM363194	0	4.5	44	M	AVR-CC	high	high	Y	II	B	2	small
GSM363204	1	14.1	53	M	CC	high	high	Y	IIIA	C	2	large
GSM363220	0	54.2	59	M	AVR-CC	high	high	Y	IIIA	B	2	large
GSM363235	0	32.8	41	M	CC	low	high	Y	III	B	2	large
GSM363251	1	19.6	47	M	CC	high	low	Y	IIIA	C	2	small
GSM363263	1	3	39	M	AVR-	high	high	Y	IIIA	C	2	large

					CC							
GSM363272	0	56.3	65	M	CC	low	high	Y	II	A	2	large
GSM363274	1	26.9	53	M	CC	high	high	Y	II	B	2	small
GSM363294	0	62.6	25	M	AVR-CC	low	high	Y	II	A	2	small
GSM363296	1	27.5	52	F	AVR-CC	low	high	Y	II	A	2	small
GSM363314	0	57.1	48	F	AVR-CC	low	high	N	II	A	2	small
GSM363326	1	37.2	37	M	CC	high	high	Y	I	A	2	small
GSM363386	1	10	61	M	CC	low	high	Y	IIIA	C	2	large
GSM712542	1	7.5	61	M	CC	low	high	Y	IIIA	C	2	small
GSM363071	1	13.8	35	M	CC	low	high	Y	IIIB	C	3	large
GSM363076	1	23	35	M	CC	high	high	Y	IIIB	C	3	large
GSM363121	0	51.2	39	M	CC	high	high	Y	IIIC	C	3	small
GSM363129	1	3.5	34	M	CC	low	high	Y	IIIA	C	3	large
GSM363211	1	8.9	61	M	CC	high	high	Y	IIIA	C	3	large
GSM363218	1	15.2	48	M	CC	low	high	Y	IIIB	A	3	large
GSM363245	0	57.5	47	M	CC	high	high	Y	IIIA	C	3	large
GSM363295	0	54.9	63	M	CC	low	high	Y	I	C	3	large
GSM363393	1	4	46	M	AVR-CC	low	high	Y	IIIA	C	3	small
GSM363100	1	3.4	56	M	CC	low	high	Y	IIIB	C	4	large
GSM363152	1	8.3	39	M	CC	high	high	Y	IIIA	C	4	large
GSM363404	1	12	46	M	CC	low	high	Y	IIIA	C	4	large
GSM363080	1	2.3	49	M	CC	low	high	Y	IIIB	C	5	large
GSM363070	0	57	50	M		high	high	Y				large
GSM363228												

GSM363334										
GSM363368	1	15.4	66	M		high	high	Y		small
GSM712532										
GSM712534										
**TANRIC (test cohort-2)**										
TCGA-BC-4072	1	49.7	74	F	white				IIIA	
TCGA-BC-4073	0	28.3	73	M	white				IIIA	
TCGA-BC-A10Q	1	37.8	72	F	white					
TCGA-BC-A10R	1	10.3	67	F	white					
TCGA-BC-A10S	1	47.4	82	M	white					
TCGA-BC-A10T	1	27.9	77	M	white					
TCGA-BC-A10U	1	27.9	69	M	white					
TCGA-BC-A10W	1	3.0	51	M	asian					
TCGA-BC-A10X	1	25.7	52	F	white				IIIA	
TCGA-BC-A10Y	1	23.7	77	M	white					
TCGA-BC-A10Z	1	1.1	63	F	white				I	

TCGA-BC-A110	1	70.5	52	F	black or african american	
TCGA-BC-A112	1	5.1	81	M	white	
TCGA-BC-A216	0	45.0	63	F	white	IIIA
TCGA-BC-A217	1	46.6	75	F	white	II
TCGA-BC-A3KF	0	0.3	67	F	white	I
TCGA-BC-A3KG	0	22.7	69	F	white	II
TCGA-BC-A5W4	1	18.2	70	M	white	IIIA
TCGA-BC-A69H	0	14.8	64	M	white	II
TCGA-BC-A69I	0	12.9	70	M	white	I
TCGA-BD-A2L6	0	45.4	70	M	white	
TCGA-BD-A3EP	0	13.6	76	F	black or african american	I
TCGA-BD-A3ER	0	37.2	62	M	white	II
TCGA-BW-A5NO	0	0.7	51	M	black or african american	IIIA
TCGA-BW-A5NP	0	0.0	27	F	white	IV

TCGA-BW-A5NQ	0	0.0	64	M	white	I
TCGA-CC-5258	1	4.3	48	M	asian	II
TCGA-CC-5259	0	8.3	60	F	asian	IIIC
TCGA-CC-5260	1	2.9	61	F	asian	IIIC
TCGA-CC-5261	1	3.2	45	M	asian	II
TCGA-CC-5262	1	3.4	68	M	asian	IIIC
TCGA-CC-5263	1	4.3	35	M	asian	IIIA
TCGA-CC-5264	1	3.4	71	M	asian	IIIA
TCGA-CC-A1HT	1	3.4	51	M	asian	IIIA
TCGA-CC-A3M9	1	10.0	45	M	asian	IIIA
TCGA-CC-A3MA	1	10.1	61	M	asian	IIIA
TCGA-CC-A3MB	1	10.5	36	M	asian	IIIA
TCGA-CC-A3MC	0	12.1	55	M	asian	IIIA
TCGA-CC-A5UC	1	11.6	63	M	asian	IIIA

TCGA-CC-A5UD	1	10.1	46	M	asian	IIIA
TCGA-CC-A5UE	1	9.1	49	M	asian	IIIB
TCGA-CC-A7IF	1	21.6	60	M	asian	IIIA
TCGA-CC-A7IG	1	10.0	48	M	asian	II
TCGA-CC-A7IH	0	12.2	59	M	asian	IIIA
TCGA-CC-A7II	0	13.3	55	M	asian	IIIA
TCGA-CC-A7IJ	0	12.7	57	M	asian	II
TCGA-CC-A7IK	1	8.7	59	M	asian	IIIA
TCGA-CC-A7IL	1	9.3	61	M	asian	IIIA
TCGA-DD-A113	0	80.8	55	F	white	II
TCGA-DD-A114	1	38.3	42	M	black or african american	II
TCGA-DD-A115	1	84.7	54	M	white	IIIA
TCGA-DD-A116	1	54.1	68	M	asian	IIIA
TCGA-DD-A118	0	114.6	78	F	white	II

TCGA-DD-A119	1	7.4	40	M	asian	IV
TCGA-DD-A11A	0	2.6	68	M	black or african american	I
TCGA-DD-A11B	1	0.5	74	M	white	I
TCGA-DD-A11C	0	22.1	70	M	white	I
TCGA-DD-A11D	1	52.0	58	F	white	I
TCGA-DD-A1EA	0	80.5	68	M	asian	II
TCGA-DD-A1EB	0	67.2	73	F	not reported	I
TCGA-DD-A1EC	0	20.1	20	F	white	I
TCGA-DD-A1ED	0	76.7	69	M	white	I
TCGA-DD-A1EE	1	11.6	74	M	white	IIIA
TCGA-DD-A1EF	1	13.1	58	F	white	I
TCGA-DD-A1EG	1	45.7	77	M	white	I
TCGA-DD-A1EH	0	49.8	23	M	white	IIi
TCGA-DD-A1	0	6.1	47	M	asian	I

EI						
TCGA-DD-A1EJ	1	33.5	71	F	white	IIIC
TCGA-DD-A1EK	1	18.6	64	F	white	IVB
TCGA-DD-A1EL	1	13.8	23	M	black or african american	II
TCGA-DD-A39V	1	21.4	77	M	white	II
TCGA-DD-A39W	1	27.6	29	F	white	IIi
TCGA-DD-A39X	1	56.5	78	F	white	I
TCGA-DD-A39Y	1	5.7	68	M	asian	I
TCGA-DD-A39Z	1	20.0	44	F	not reported	II
TCGA-DD-A3A1	1	7.8	66	M	not reported	IIIA
TCGA-DD-A3A2	1	71.0	77	F	white	I
TCGA-DD-A3A3	1	17.8	46	M	asian	I
TCGA-DD-A3A4	1	20.4	38	M	white	IIIA
TCGA-DD-A3	1	104.2	67	F	white	IIi

A5						
TCGA-DD-A3A6	1	108.6	72	F	white	II
TCGA-DD-A3A7	1	14.0	67	M	not reported	IIIB
TCGA-DD-A3A8	1	0.4	75	M	white	II
TCGA-DD-A3A9	1	31.0	64	F	white	IVB
TCGA-DD-A4NA	0	33.6	67	F	white	IIIC
TCGA-DD-A4NB	0	33.0	26	M	white	I
TCGA-DD-A4ND	0	91.5	57	F	white	I
TCGA-DD-A4NE	1	22.0	75	F	white	IIIA
TCGA-DD-A4NF	0	31.4	72	M	white	I
TCGA-DD-A4NG	1	14.8	77	M	white	IIIA
TCGA-DD-A4NH	0	30.6	65	F	white	IIIB
TCGA-DD-A4NI	0	27.2	67	M	white	II
TCGA-DD-A4NJ	0	30.9	55	F	white	II

TCGA-DD-A4NK	1	40.3	80	F	white	IIIA
TCGA-DD-A4NL	0	57.0	46	M	white	I
TCGA-DD-A4NN	1	30.0	56	F	white	I
TCGA-DD-A4NO	0	74.8	66	M	white	I
TCGA-DD-A4NP	0	110.3	32	M	white	I
TCGA-DD-A4NQ	1	12.4	61	M	white	II
TCGA-DD-A4NR	1	0.3	86	F	white	I
TCGA-DD-A4NS	1	81.9	62	F	white	I
TCGA-DD-A4NV	0	79.9	61	M	white	IIIA
TCGA-DD-A73A	0	24.3	72	M	white	I
TCGA-DD-A73B	1	9.4	72	F	white	I
TCGA-DD-A73C	0	23.4	65	F	white	IIIA
TCGA-DD-A73D	0	23.1	68	F	white	II
TCGA-DD-A73E	0	1.5	67	M	white	I

TCGA-DD-A73F	0	36.2	77	F	white	I
TCGA-DD-A73G	0	115.9	74	F	white	I
TCGA-ED-A459	0	30.3	48	M	asian	II
TCGA-ED-A4XI	0	27.3	59	M	asian	II
TCGA-ED-A5KG	0	28.5	61	F	asian	II
TCGA-ED-A627	0	14.1	75	M	white	I
TCGA-ED-A66X	0	13.5	35	M	asian	IIIA
TCGA-ED-A66Y	1	9.9	51	F	asian	IIIA
TCGA-ED-A7PX	0	0.2	48	F	asian	II
TCGA-ED-A7PY	0	13.0	21	F	asian	II
TCGA-ED-A7PZ	0	0.2	62	M	asian	II
TCGA-ED-A7XP	0	13.3	53	F	asian	II
TCGA-ED-A82E	0	13.6	61	F	asian	IIIA
TCGA-EP-A12J	0	19.0	63	M	black or african american	I

TCGA-EP-A26S	0	20.3	70	M	white	I
TCGA-EP-A2KA	1	11.9	53	F	white	IIIA
TCGA-EP-A2KB	1	11.1	46	F	white	I
TCGA-EP-A2KC	1	0.6	62	M	black or african american	I
TCGA-EP-A3JL	0	10.1	76	M	white	I
TCGA-EP-A3RK	0	12.1	74	M	white	IIIA
TCGA-ES-A2HS	1	22.9	80	M	white	I
TCGA-ES-A2HT	1	14.6	55	M	black or african american	I
TCGA-FV-A23B	1	61.7	71	F	white	II
TCGA-FV-A2QQ	0	24.3	80	M	white	I
TCGA-FV-A2QR	1	19.4		M	white	I
TCGA-FV-A3I0	0	28.3	77	F	white	II
TCGA-FV-A3I1	1	8.2		F	white	II
TCGA-FV-A3R2	1	6.5	75	M	white	I

TCGA-FV-A3R3	1	12.2	39	F	white	I
TCGA-FV-A495	0	0.0	51	F	white	II
TCGA-FV-A496	0	0.3	85	F	white	I
TCGA-FV-A4ZP	1	82.9	79	M	white	IIIA
TCGA-FV-A4ZQ	0	0.4	52	M	white	I
TCGA-G3-A25S	1	13.9	65	M	white	I
TCGA-G3-A25T	0	51.8	45	F	white	IIIA
TCGA-G3-A25U	0	54.5	64	F	asian	I
TCGA-G3-A25V	0	28.7	69	M	white	I
TCGA-G3-A25X	0	59.3	74	M	asian	II
TCGA-G3-A25Y	1	15.1	52	F	asian	I
TCGA-G3-A25Z	0	21.8	59	M	asian	I
TCGA-G3-A3CG	0	22.4	81	M	white	I
TCGA-G3-A3CH	0	26.0	53	M	asian	IIIA

TCGA-G3-A3CI	0	6.0	72	M	white	I
TCGA-G3-A3CJ	0	19.8	53	M	american indian or alaska native	II
TCGA-G3-A3CK	0	19.5	62	M	asian	I
TCGA-G3-A5SI	1	25.6	44	M	asian	II
TCGA-G3-A5SJ	0	23.3	60	M	white	I
TCGA-G3-A5SK	0	24.8	58	M	white	I
TCGA-G3-A5SL	0	20.7	70	M	white	II
TCGA-G3-A5SM	0	17.3	58	M	white	II
TCGA-G3-A6UC	0	22.4	65	M	white	IIIB
TCGA-G3-A7M5	0	14.9	77	M	asian	I
TCGA-G3-A7M6	0	21.1	61	F	white	I
TCGA-G3-A7M7	0	12.0	66	M	white	I
TCGA-G3-A7M8	0	14.3	32	M	asian	I
TCGA-G3-A7M9	1	1.9	70	M	white	IIIB

TCGA-GJ-A6C0	1	1.0	76	F	white	II
TCGA-HP-A5MZ	1	3.0	62	M	not reported	I
TCGA-HP-A5N0	1	38.2	88	F	not reported	
TCGA-K7-A5RF	0	21.0	65	M	white	I
TCGA-K7-A5RG	0	17.3	66	M	black or african american	I
TCGA-K7-A6G5	0	17.1	66	M	white	I
TCGA-KR-A7K0	1	2.2	66	M	white	I
TCGA-KR-A7K2	0	27.6	65	M	white	I
TCGA-KR-A7K7	0	31.7	62	F	white	II
TCGA-KR-A7K8	0	30.2	57	M	not reported	I
TCGA-LG-A6GG	0	12.9	79	F	white	II
TCGA-MI-A75C	0	9.7	64	M	white	I
TCGA-MI-A75	0	16.9	62	M	white	IIIC

E						
TCGA-MI-A75G	0	23.3	64	M	white	II
TCGA-MI-A75H	0	24.9	78	M	white	
TCGA-MI-A75I	0	21.0	61	M	black or african american	
TCGA-MR-A520	0	7.6	59	M	white	I
TCGA-NI-A4U2	1	59.7	71	M	white	IIIA
TCGA-O8-A75V	0	17.9	54	M	not reported	I
TCGA-PD-A5DF	1	21.3	59	F	white	IIIB
TCGA-QA-A7B7	0	3.1	48	M	black or african american	II
TCGA-RC-A6M3	0	0.0	24	M	american indian or alaska native	II
TCGA-RC-A6M4	0	0.7	75	F	white	IIIA
TCGA-RC-A6M5	0	0.5	21	F	white	IVA
TCGA-RC-A6M6	0	0.3	76	M	white	II
TCGA-RC-A7S9	0	21.3	48	F	asian	I

TCGA-RC-A7SB	0	19.6	54	M	asian	II
TCGA-RC-A7SF	0	19.3	67	M	asian	I
TCGA-RC-A7SK	0	15.7	60	M	asian	I
TCGA-RG-A7D4	0	36.6	69	M	black or african american	II
TCGA-T1-A6J8	0	0.8	68	M	white	
TCGA-UB-A7MA	0	28.3	63	F	white	II
TCGA-UB-A7MB	0	20.0	25	M	white	II
TCGA-UB-A7MC	0	16.7	60	M	white	IIIA
TCGA-UB-A7MD	1	1.7	68	M	black or african american	I
TCGA-UB-A7ME	0	16.2	52	M	asian	I
TCGA-UB-A7MF	1	7.1	57	M	white	IIIA

[1]Coding: 0, survival, and 1, death.

[2]M, male, and F, female.

[3]CC, chronic carrier, AVR-CC, active viral replication chronic carrier, and N, normal.

[4]Y, yes, and N, no.

Table S2. GO term and KEGG pathway enrichment analysis of mRNAs correlated with the four lncRNAs in the prediction signature.

GO terms and KEGG pathways	NO. of genes	*P*-value	Fold Enrichment
**GO terms**			
GO:0006614~SRP-dependent cotranslational protein targeting to membrane	70	8.03E-52	8.253915
GO:0006413~translational initiation	84	9.96E-52	6.795924
GO:0006364~rRNA processing	104	6.03E-51	5.386533
GO:0019083~viral transcription	73	1.29E-47	7.224281
GO:0000184~nuclear-transcribed mRNA catabolic process, nonsense-mediated decay	73	6.11E-45	6.799323
GO:0006412~translation	102	7.26E-41	4.468579
GO:0000398~mRNA splicing, via spliceosome	70	5.68E-21	3.494901
GO:0070126~mitochondrial translational termination	30	1.61E-10	3.866452
GO:0070125~mitochondrial translational elongation	29	6.15E-10	3.781541
GO:0000387~spliceosomal snRNP assembly	15	2.64E-08	5.937765
GO:0000027~ribosomal large subunit assembly	12	4.65E-07	6.333616
GO:0008380~RNA splicing	36	1.55E-06	2.403722
GO:0006626~protein targeting to mitochondrion	14	3.76E-06	4.563929
GO:0002181~cytoplasmic translation	12	4.18E-06	5.320238
GO:0000245~spliceosomal complex assembly	12	6.66E-06	5.115613
GO:0042274~ribosomal small subunit biogenesis	9	2.85E-05	6.234653
GO:0033344~cholesterol efflux	11	3.18E-05	4.876884
GO:0000462~maturation of SSU-rRNA from tricistronic rRNA transcript (SSU-rRNA, 5.8S rRNA, LSU-rRNA)	12	6.69E-05	4.156436
GO:0051170~nuclear import	9	8.22E-05	5.541914
GO:0006953~acute-phase response	13	0.000107	3.694609
GO:0000028~ribosomal small subunit assembly	9	0.000131	5.250234
GO:0031145~anaphase-promoting complex-dependent catabolic process	19	0.000186	2.665731
GO:0032543~mitochondrial translation	12	0.000222	3.694609
GO:0010467~gene expression	14	0.000236	3.232783
GO:0006283~transcription-coupled nucleotide-excision repair	18	0.000251	2.696066
GO:0070328~triglyceride homeostasis	10	0.000291	4.263011
GO:0006699~bile acid biosynthetic process	9	0.000299	4.750212
GO:0045039~protein import into mitochondrial inner membrane	5	0.000306	11.08383
GO:0042157~lipoprotein metabolic process	12	0.000376	3.500156
GO:0016032~viral process	46	0.000438	1.705204
GO:0042632~cholesterol homeostasis	16	0.000453	2.770957
GO:0010873~positive regulation of cholesterol	6	0.000547	7.389219

esterification			
GO:0043691~reverse cholesterol transport	8	0.000623	4.926146
GO:0098609~cell-cell adhesion	42	0.000694	1.717789
GO:0043488~regulation of mRNA stability	21	0.00079	2.25981
GO:0033700~phospholipid efflux	7	0.000846	5.541914
GO:0034384~high-density lipoprotein particle clearance	5	0.000851	9.236524
GO:0090502~RNA phosphodiester bond hydrolysis, endonucleolytic	14	0.00098	2.821338
GO:0006446~regulation of translational initiation	11	0.000985	3.386725
GO:0042273~ribosomal large subunit biogenesis	9	0.001145	3.990178
GO:0034375~high-density lipoprotein particle remodeling	7	0.001302	5.172453
GO:0051436~negative regulation of ubiquitin-protein ligase activity involved in mitotic cell cycle	16	0.001428	2.497764
GO:0006094~gluconeogenesis	12	0.00145	3.022862
GO:0006631~fatty acid metabolic process	13	0.001892	2.770957
GO:0002576~platelet degranulation	20	0.001982	2.1522
GO:0017144~drug metabolic process	9	0.001998	3.694609
GO:0008543~fibroblast growth factor receptor signaling pathway	17	0.002412	2.297867
GO:0000244~spliceosomal tri-snRNP complex assembly	6	0.00273	5.541914
GO:0008334~histone mRNA metabolic process	6	0.00273	5.541914
GO:0030150~protein import into mitochondrial matrix	7	0.002751	4.563929
GO:0051437~positive regulation of ubiquitin-protein ligase activity involved in regulation of mitotic cell cycle transition	16	0.002896	2.333438
GO:0006457~protein folding	29	0.003049	1.785728
GO:0006641~triglyceride metabolic process	10	0.003134	3.166808
GO:0001731~formation of translation preinitiation complex	8	0.003212	3.855245
GO:0060071~Wnt signaling pathway, planar cell polarity pathway	18	0.003273	2.168575
GO:0050434~positive regulation of viral transcription	9	0.003284	3.439809
GO:0043928~exonucleolytic nuclear-transcribed mRNA catabolic process involved in deadenylation-dependent decay	9	0.003284	3.439809
GO:0006120~mitochondrial electron transport, NADH to ubiquinone	12	0.003629	2.714407
GO:0006414~translational elongation	7	0.003815	4.310378
GO:0042254~ribosome biogenesis	10	0.00385	3.078841
GO:0006183~GTP biosynthetic process	6	0.00411	5.115613
GO:0045116~protein neddylation	6	0.00411	5.115613
GO:0008033~tRNA processing	10	0.004688	2.995629
GO:0017148~negative regulation of translation	13	0.004944	2.484306
GO:0007067~mitotic nuclear division	36	0.005229	1.608943

GO:0038061~NIK/NF-kappaB signaling	14	0.005458	2.351115
GO:0006396~RNA processing	18	0.005722	2.056793
GO:0097267~omega-hydroxylase P450 pathway	5	0.005726	6.157682
GO:0038033~positive regulation of endothelial cell chemotaxis by VEGF-activated vascular endothelial growth factor receptor signaling pathway	4	0.006364	8.867063
GO:0006370~7-methylguanosine mRNA capping	9	0.007691	3.022862
GO:0000470~maturation of LSU-rRNA	6	0.008235	4.433531
GO:1904874~positive regulation of telomerase RNA localization to Cajal body	6	0.008235	4.433531
GO:0042730~fibrinolysis	7	0.008823	3.694609
GO:0048025~negative regulation of mRNA splicing, via spliceosome	7	0.008823	3.694609
GO:0042776~mitochondrial ATP synthesis coupled proton transport	7	0.008823	3.694609
GO:0007084~mitotic nuclear envelope reassembly	5	0.008867	5.541914
GO:0006367~transcription initiation from RNA polymerase II promoter	24	0.009468	1.750078
GO:0032981~mitochondrial respiratory chain complex I assembly	13	0.009748	2.287139
GO:0034382~chylomicron remnant clearance	4	0.011877	7.389219
GO:0006754~ATP biosynthetic process	8	0.012695	3.057608
GO:0006098~pentose-phosphate shunt	5	0.012952	5.038104
GO:0001682~tRNA 5'-leader removal	5	0.012952	5.038104
GO:0008202~steroid metabolic process	10	0.013039	2.577635
GO:0000413~protein peptidyl-prolyl isomerization	10	0.013039	2.577635
GO:0006521~regulation of cellular amino acid metabolic process	11	0.014377	2.39063
GO:0006069~ethanol oxidation	5	0.018063	4.618262
GO:0006228~UTP biosynthetic process	5	0.018063	4.618262
GO:0006241~CTP biosynthetic process	5	0.018063	4.618262
GO:0036258~multivesicular body assembly	8	0.018267	2.860343
GO:0006351~transcription, DNA-templated	203	0.018709	1.150904
GO:0007597~blood coagulation, intrinsic pathway	6	0.018723	3.694609
GO:0019373~epoxygenase P450 pathway	6	0.018723	3.694609
GO:0006165~nucleoside diphosphate phosphorylation	6	0.018723	3.694609
GO:0075522~IRES-dependent viral translational initiation	4	0.019403	6.333616
GO:0046689~response to mercury ion	4	0.019403	6.333616
GO:0006465~signal peptide processing	7	0.021119	3.103472
GO:0046686~response to cadmium ion	7	0.021119	3.103472
GO:1903955~positive regulation of protein targeting to mitochondrion	15	0.021217	1.933226

GO:0006805~xenobiotic metabolic process	14	0.021592	1.989405
GO:0032212~positive regulation of telomere maintenance via telomerase	8	0.021613	2.770957
GO:0034370~triglyceride-rich lipoprotein particle remodeling	3	0.02291	11.08383
GO:0010903~negative regulation of very-low-density lipoprotein particle remodeling	3	0.02291	11.08383
GO:0051301~cell division	44	0.022932	1.393396
GO:0036109~alpha-linolenic acid metabolic process	5	0.024264	4.263011
GO:0006957~complement activation, alternative pathway	5	0.024264	4.263011
GO:0033540~fatty acid beta-oxidation using acyl-CoA oxidase	5	0.024264	4.263011
GO:0006418~tRNA aminoacylation for protein translation	9	0.024323	2.493861
GO:1903146~regulation of mitophagy	8	0.025362	2.686989
GO:0006383~transcription from RNA polymerase III promoter	8	0.025362	2.686989
GO:0045471~response to ethanol	17	0.025721	1.794525
GO:0043161~proteasome-mediated ubiquitin-dependent protein catabolic process	28	0.02631	1.528804
GO:0008152~metabolic process	24	0.028442	1.583404
GO:0048261~negative regulation of receptor-mediated endocytosis	4	0.02899	5.541914
GO:0034380~high-density lipoprotein particle assembly	4	0.02899	5.541914
GO:0034475~U4 snRNA 3'-end processing	4	0.02899	5.541914
GO:0010898~positive regulation of triglyceride catabolic process	4	0.02899	5.541914
GO:0072321~chaperone-mediated protein transport	4	0.02899	5.541914
GO:0075713~establishment of integrated proviral latency	4	0.02899	5.541914
GO:0010269~response to selenium ion	4	0.02899	5.541914
GO:0032091~negative regulation of protein binding	11	0.029811	2.138984
GO:0006400~tRNA modification	7	0.030264	2.873585
GO:0006355~regulation of transcription, DNA-templated	157	0.03154	1.157022
GO:0007584~response to nutrient	13	0.032267	1.947159
GO:0006310~DNA recombination	14	0.034222	1.869561
GO:0061418~regulation of transcription from RNA polymerase II promoter in response to hypoxia	7	0.035675	2.770957
GO:0043966~histone H3 acetylation	9	0.036145	2.319871
GO:0090090~negative regulation of canonical Wnt signaling pathway	23	0.036281	1.563976
GO:0016236~macroautophagy	13	0.038705	1.895918
GO:0007017~microtubule-based process	8	0.039199	2.463073
GO:0042769~DNA damage response, detection of DNA	8	0.039199	2.463073

damage			
GO:0006388~tRNA splicing, via endonucleolytic cleavage and ligation	5	0.040096	3.694609
GO:0048026~positive regulation of mRNA splicing, via spliceosome	5	0.040096	3.694609
GO:0042158~lipoprotein biosynthetic process	4	0.040622	4.926146
GO:0051569~regulation of histone H3-K4 methylation	4	0.040622	4.926146
GO:0034427~nuclear-transcribed mRNA catabolic process, exonucleolytic, 3'-5'	4	0.040622	4.926146
GO:0006220~pyrimidine nucleotide metabolic process	4	0.040622	4.926146
GO:0015825~L-serine transport	4	0.040622	4.926146
GO:0071028~nuclear mRNA surveillance	4	0.040622	4.926146
GO:0070475~rRNA base methylation	4	0.040622	4.926146
GO:0006635~fatty acid beta-oxidation	9	0.040818	2.267147
GO:0051289~protein homotetramerization	11	0.040829	2.032035
GO:0006637~acyl-CoA metabolic process	6	0.042648	3.022862
GO:0034314~Arp2/3 complex-mediated actin nucleation	6	0.042648	3.022862
GO:0034227~tRNA thio-modification	3	0.04309	8.312871
GO:0010916~negative regulation of very-low-density lipoprotein particle clearance	3	0.04309	8.312871
GO:0036101~leukotriene B4 catabolic process	3	0.04309	8.312871
GO:0048386~positive regulation of retinoic acid receptor signaling pathway	3	0.04309	8.312871
GO:0032375~negative regulation of cholesterol transport	3	0.04309	8.312871
GO:0045833~negative regulation of lipid metabolic process	3	0.04309	8.312871
GO:0032447~protein urmylation	3	0.04309	8.312871
GO:0031118~rRNA pseudouridine synthesis	3	0.04309	8.312871
GO:0006368~transcription elongation from RNA polymerase II promoter	14	0.043959	1.804344
GO:0006338~chromatin remodeling	14	0.043959	1.804344
GO:0007080~mitotic metaphase plate congression	8	0.044726	2.396503
GO:0006091~generation of precursor metabolites and energy	10	0.045837	2.091288
GO:0042795~snRNA transcription from RNA polymerase II promoter	12	0.047899	1.900085
GO:0030449~regulation of complement activation	7	0.048262	2.586227
GO:0033574~response to testosterone	7	0.048262	2.586227
GO:0043252~sodium-independent organic anion transport	6	0.050591	2.891433
GO:0006888~ER to Golgi vesicle-mediated transport	22	0.05162	1.524026
GO:0006397~mRNA processing	24	0.052867	1.4861
GO:0033619~membrane protein proteolysis	4	0.054231	4.433531
GO:0006163~purine nucleotide metabolic process	4	0.054231	4.433531

GO:0006000~fructose metabolic process	4	0.054231	4.433531
GO:0045292~mRNA cis splicing, via spliceosome	4	0.054231	4.433531
GO:0051918~negative regulation of fibrinolysis	4	0.054231	4.433531
GO:0016192~vesicle-mediated transport	21	0.055258	1.531318
GO:0019058~viral life cycle	7	0.055461	2.5028
GO:0006297~nucleotide-excision repair, DNA gap filling	6	0.059342	2.770957
GO:0006369~termination of RNA polymerase II transcription	11	0.059445	1.905033
GO:0021987~cerebral cortex development	10	0.061712	1.979255
GO:0015918~sterol transport	3	0.067575	6.650297
GO:0030300~regulation of intestinal cholesterol absorption	3	0.067575	6.650297
GO:0002192~IRES-dependent translational initiation	3	0.067575	6.650297
GO:0033599~regulation of mammary gland epithelial cell proliferation	3	0.067575	6.650297
GO:0019626~short-chain fatty acid catabolic process	3	0.067575	6.650297
GO:0075525~viral translational termination-reinitiation	3	0.067575	6.650297
GO:0051005~negative regulation of lipoprotein lipase activity	3	0.067575	6.650297
GO:0046822~regulation of nucleocytoplasmic transport	3	0.067575	6.650297
GO:0048254~snoRNA localization	3	0.067575	6.650297
GO:0032489~regulation of Cdc42 protein signal transduction	3	0.067575	6.650297
GO:0050996~positive regulation of lipid catabolic process	3	0.067575	6.650297
GO:0019886~antigen processing and presentation of exogenous peptide antigen via MHC class II	14	0.06889	1.68667
GO:0000375~RNA splicing, via transesterification reactions	6	0.068897	2.660119
GO:0015914~phospholipid transport	6	0.068897	2.660119
GO:0019433~triglyceride catabolic process	6	0.068897	2.660119
GO:0034374~low-density lipoprotein particle remodeling	4	0.069711	4.030483
GO:0046135~pyrimidine nucleoside catabolic process	4	0.069711	4.030483
GO:0017187~peptidyl-glutamic acid carboxylation	4	0.069711	4.030483
GO:0006071~glycerol metabolic process	4	0.069711	4.030483
GO:1902188~positive regulation of viral release from host cell	4	0.069711	4.030483
GO:0016197~endosomal transport	11	0.070537	1.847305
GO:0043623~cellular protein complex assembly	5	0.072545	3.078841
GO:0033539~fatty acid beta-oxidation using acyl-CoA dehydrogenase	5	0.072545	3.078841
GO:0007004~telomere maintenance via telomerase	5	0.072545	3.078841
GO:0010951~negative regulation of endopeptidase activity	17	0.077204	1.557232
GO:0006281~DNA repair	29	0.077823	1.367792
GO:0007062~sister chromatid cohesion	15	0.078649	1.61415

GO:0046487~glyoxylate metabolic process	6	0.079249	2.557807
GO:0043248~proteasome assembly	4	0.086927	3.694609
GO:0010458~exit from mitosis	4	0.086927	3.694609
GO:0000050~urea cycle	4	0.086927	3.694609
GO:0019068~virion assembly	4	0.086927	3.694609
GO:0042738~exogenous drug catabolic process	4	0.086927	3.694609
GO:0034260~negative regulation of GTPase activity	8	0.088082	2.062108
GO:0042594~response to starvation	7	0.090373	2.216766
GO:0043985~histone H4-R3 methylation	3	0.095423	5.541914
GO:0006066~alcohol metabolic process	3	0.095423	5.541914
GO:0070508~cholesterol import	3	0.095423	5.541914
GO:1902231~positive regulation of intrinsic apoptotic signaling pathway in response to DNA damage	3	0.095423	5.541914
GO:0042304~regulation of fatty acid biosynthetic process	3	0.095423	5.541914
GO:0061154~endothelial tube morphogenesis	3	0.095423	5.541914
GO:0033119~negative regulation of RNA splicing	3	0.095423	5.541914
GO:0010501~RNA secondary structure unwinding	8	0.097023	2.015242
GO:0034644~cellular response to UV	8	0.097023	2.015242
GO:0032508~DNA duplex unwinding	8	0.097023	2.015242
GO:0009813~flavonoid biosynthetic process	5	0.099719	2.770957
GO:0006123~mitochondrial electron transport, cytochrome c to oxygen	5	0.099719	2.770957
**KEGG pathways**			
hsa03010:Ribosome	86	6.16E-51	6.137021
hsa03040:Spliceosome	42	3.9E-11	3.064755
hsa04610:Complement and coagulation cascades	24	1.65E-07	3.375672
hsa01100:Metabolic pathways	174	1.96E-06	1.375146
hsa05016:Huntington's disease	41	8.71E-06	2.072434
hsa00071:Fatty acid degradation	16	1.75E-05	3.529111
hsa04932:Non-alcoholic fatty liver disease (NAFLD)	31	0.000279	1.992429
hsa00240:Pyrimidine metabolism	24	0.000286	2.239628
hsa03320:PPAR signaling pathway	18	0.000322	2.607329
hsa01200:Carbon metabolism	25	0.000404	2.147136
hsa01130:Biosynthesis of antibiotics	38	0.000836	1.739586
hsa03008:Ribosome biogenesis in eukaryotes	20	0.001109	2.231047
hsa03013:RNA transport	32	0.001262	1.805592
hsa00190:Oxidative phosphorylation	26	0.001995	1.897229
hsa04146:Peroxisome	18	0.004056	2.104711
hsa00630:Glyoxylate and dicarboxylate metabolism	9	0.004531	3.235019
hsa05012:Parkinson's disease	26	0.004939	1.776982
hsa00120:Primary bile acid biosynthesis	7	0.005317	3.9962
hsa00983:Drug metabolism - other enzymes	12	0.005836	2.531754

hsa00010:Glycolysis / Gluconeogenesis	15	0.00726	2.172774
hsa00982:Drug metabolism - cytochrome P450	15	0.008304	2.140821
hsa00410:beta-Alanine metabolism	9	0.011135	2.817597
hsa04962:Vasopressin-regulated water reabsorption	11	0.012145	2.426264
hsa03020:RNA polymerase	9	0.013544	2.729547
hsa00650:Butanoate metabolism	8	0.016775	2.875572
hsa01230:Biosynthesis of amino acids	15	0.017281	1.967241
hsa00280:Valine, leucine and isoleucine degradation	11	0.019149	2.271396
hsa00640:Propanoate metabolism	8	0.020439	2.772873
hsa03018:RNA degradation	15	0.0239	1.890595
hsa03030:DNA replication	9	0.027039	2.426264
hsa00830:Retinol metabolism	13	0.03141	1.941011
hsa03050:Proteasome	10	0.03244	2.205695
hsa05010:Alzheimer's disease	26	0.036752	1.501973
hsa04976:Bile secretion	13	0.047223	1.828489
hsa04931:Insulin resistance	18	0.048805	1.617509
hsa00053:Ascorbate and aldarate metabolism	7	0.052547	2.516126
hsa00230:Purine metabolism	26	0.058822	1.433701
hsa05204:Chemical carcinogenesis	14	0.063585	1.698385
hsa00040:Pentose and glucuronate interconversions	8	0.070598	2.156679
hsa00030:Pentose phosphate pathway	7	0.070872	2.3426
hsa04152:AMPK signaling pathway	19	0.073503	1.511443
hsa04122:Sulfur relay system	4	0.075124	3.882022
hsa02010:ABC transporters	9	0.076928	1.985125
hsa04922:Glucagon signaling pathway	16	0.080487	1.568494
hsa03015:mRNA surveillance pathway	15	0.080729	1.599735
hsa04961:Endocrine and other factor-regulated calcium reabsorption	9	0.085562	1.941011
hsa04975:Fat digestion and absorption	8	0.099911	1.990781
hsa00260:Glycine, serine and threonine metabolism	8	0.099911	1.990781
